# Effects of a compound *Trichoderma* agent on *Coptis chinensis* growth, nutrients, enzyme activity, and microbial community of rhizosphere soil

**DOI:** 10.7717/peerj.15652

**Published:** 2023-07-12

**Authors:** Li X. Wu, Yu Wang, Hui Lyu, Xia D. Chen

**Affiliations:** 1 Institute of Material Medical Planting, Chongqing Academy of Chinese Materia Medica, Chongqing, China; 2 Chongqing Engineering Research Center for Fine Variety Breeding Techniques of Chinese Materia Medica, Chongqing, China; 3 Chongqing Key Laboratory of Traditional Chinese Medicine Resource, Chongqing, China; 4 Chongqing Sub-center of National Resource Center for Chinese Materia Medica, China Academy of Chinese Medical Science, Chongqing, China

**Keywords:** Compound *Trichoderma* agent, *Coptis chinensis* growth, Rhizosphere soil, Nutrients, Enzyme activity, Microbial community

## Abstract

**Background:**

Root rot diseases are prevalent in many *Coptis chinensis* Franch. production areas, perhaps partially due to the overuse of synthetic fertilizers. Synthetic fertilizers can also lead to soil degradation. *Trichoderma* is widely used in biofertilizers and biopesticides. This study applied a combination of four *Trichoderma* species (compound *Trichoderma* agent, CTA) to *C. chinensis* and evaluated its effects on growth, as well as rhizosphere soil nutrients, enzyme activities, and microbial community structure. The purpose of this study was to estimate the potential of using CTA as a biofertilizer for *C. chinensis*, and determine if it could, at least partially, replace synthetic fertilizers to control root rot disease and maintain soil fertility.

**Method:**

CTA, compound fertilizer and sterile water were applied to *C. chinensis* plants. After 60 days, the soluble sugar, soluble protein, chlorophyll of leaves, and individual weight of each plant were measured. The rhizosphere soil nutrient content, enzymatic activity, and the microbial community were also determined. The results were analyzed to evaluate the effect of CTA on *C. chinensis* growth and soil fertility.

**Results:**

CTA increased the soluble protein, chlorophyll, and individual weight of *C. chinensis* plants while compound fertilizer decreased chlorophyll. CTA increased the activities of urease and catalase in rhizosphere soil, whereas the compound fertilizer decreased urease, catalase, and alkaline phosphatase activities. CTA elevated soil pH, while compound fertilizer reduced it. CTA had no significant effects on soil nutrients and organic matter. CTA decreased the fungal number and alpha-diversity of fungi and bacteria, and both the fungal and bacterial communities were significantly different from the other two. CTA increased B/F value, which improved the rhizosphere microbial community. Both CTA and the compound fertilizer significantly altered the soil microbial community. The relative abundance of Ascomycota was higher and Basidiomycota was lower after CTA treatment than after the other two treatments, indicating that the soil treated with CTA was healthier than that of the other two treatments. CTA decreased harmful *Ilyonectria mors-panacis* and *Corynebacterium* sp. And increased beneficial *Ralstonia picketti. Trichoderma* spp. could exist in *C. chinensis* rhizosphere soil for a long time. The functional prediction results demonstrated that CTA reduced some rhizosphere phytopathogenic fungi. Correlation analysis showed that CTA elevated rhizosphere pH and enzyme activities. In summary, synthetic fertilizers damaged soil fertility, and the overuse of them might be responsible for root rot disease, while CTA could promote *C. chinensis* growth, improve soil and decrease the incidence and severity of *C. chinensis* root rot disease. Therefore, as a biofertilizer, CTA can, at least partially, replace synthetic fertilizers in *C. chinensis* production. Combining it with organic fertilizer will increase the potential of *Trichoderma*.

## Introduction

*Coptis chinensis* Franch. (*C. chinensis*) is a perennial herb belonging to the genus *Coptis* and the family Ranunculaceae. The herbal medicine made from its dried rhizomes is one of the most commonly used traditional herbal medicines in China. The Coptidis rhizome contains various chemical components including alkaloids, flavonoids, organic acids, polysaccharides, *etc*. It has hypoglycemic and hypolipidemic effects, as well as antibacterial and antitumor properties (Gai et al., 2018). In the last decade, root rot disease has broken out in the main *C. chinensis* producing areas in Sichuan, Hubei, and Chongqing, China. Root rot begins in the fibrous root and then expands to the taproot. The taproot rots and turns black, and the leaves turn red and wither. In severe cases, all *C. chinensis* plants in the entire plot will die. The economic loss to farmers can be up to US $70,000–100,000 per hectare. Many farmers even give up planting *C. chinensis*, resulting in a dramatically reduced production area, plummeted yield, and thus surging prices of Coptidis rhizome.

Although *C. chinensis* has been produced in many areas for decades or even hundreds of years, root rot disease broke out only in the last decade. One reason for this might be global warming. The increased temperature in high-altitude areas that previously had lower temperatures encouraged the growth and infection of pathogenic microorganisms (Cohen & Leach, 2020; Wu et al., 2020). For example, *Fusarium oxysporum*, one of the main pathogens responsible for *C. chinensis* root rot disease, is pathogenic only at higher temperatures (Wu et al., 2020). Another reason might be the overuse of synthetic fertilizers, such as urea, potassic fertilizer, and super phosphate. Some inorganic nutrient contents are directly associated with disease severity. It was found that the disease severity of crown and root rot of tomato caused by *F. oxysporum* was significantly increased by the levels of NH_4_Cl, (NH_4_)_2_SO_4_ and high rates of NH_4_NO_3_ (Duffy & Défago, 1999). Additionally, long-term and large-scale application of synthetic fertilizers caused soil acidification, which aggravated the occurrence of diseases (Shen et al., 2018). Due to serious continuous cropping obstacles in recent decades, *C. chinensis* is generally rotated with grains, vegetables, and fruit trees. Synthetic fertilizers are heavily applied in the production of these crops and *C. chinensis* (Hou et al., 2017), which might be responsible for the prevalence of root rot. Synthetic fertilizers also lead to soil acidification (Tang et al., 2020), soil compaction (Massah & Azadegan, 2016), and heavy metal accumulation (Niño-Savala et al., 2019), which lead to soil degradation. In summary, synthetic fertilizers should be, at least partially, replaced by environmentally-friendly ones in *C. chinensis* production.

Most *Trichoderma* spp. live in soil (Gherbawy, Hussein & Al-Qurashi, 2014). It is an important class of beneficial fungi that can promote plant growth (Yu et al., 2021); antagonize at least 29 pathogenic fungi belonging to 18 genera (Win et al., 2021) to control plant diseases (Li et al., 2018a); degrade pesticides (Sharma et al., 2016); recover heavy metals such as Cr, Cu, and Pb (Tansengco et al., 2018); boost the activities of soil enzymes (Baazeem et al., 2021); and regulate soil acidity (Jian et al., 2021). Therefore, it has been widely applied in the research and development of biofertilizers and biopesticides. When biofertilizer made from four *Trichoderma* species was applied to flowering Chinese cabbage, the yield increased by 37.4%. It also elevated the contents of soluble sugar, soluble protein, and chlorophyll. The activities of soil urease, phosphatase, and catalase increased by 25.1%, 13.1%, and 14.0%, respectively (Ji et al., 2020).

Therefore, *Trichoderma* spp. can control root rot in *C. chinensis* by: (1) directly antagonizing pathogens as a biopesticide, and (2), at least partially, possibly replacing synthetic fertilizers as biofertilizer. Additionally, it has the potential for improving soil. Our previous study revealed that an agent consisting of four *Trichoderma* species (compound *Trichoderma* agent, CTA) could decrease the incidence and severity of *C. chinensis* root rot disease by 64.00% and 59.32%, respectively, which demonstrated its potential as a biopesticide against root rot. Therefore, in this study, we examined the effects of CTA on *C. chinensis* growth and rhizosphere soil nutrients, enzyme activities, and microbial community structure in order to estimate its potential as a biofertilizer for *C. chinensis* that can, at least partially, replace synthetic fertilizer, control root rot, and maintain soil fertility.

No study has applied *Trichoderma* spp. to *C. chinensis* both as a biopesticide and biofertilizer to control root rot. Related research is scarce as well. According to Bian (2022), compared with the conventional nitrogen fertilizer treatment (CK), 25% reduction of nitrogen fertilizer in combination with *Pseudomonas fluorescence* engineering strain (which had both biocontrol activity and nitrogen-fixing function) decreased the incidence of root rot from 8.8% to 6.07% and the disease index from 3.31 to 2.72 in garlic production. The yield and allicin content increased by 10.55% and 14.29%, respectively. The results indicated that replacing synthetic fertilizer partially with biofertilizer could control root rot. This study applied this theory to *C. chinensis*. Previous studies of *C. chinensis* root rot control emphasized pesticides and paid little attention to the direct and indirect effect of synthetic fertilizers on root rot severity, while this study evaluated the feasibility of replacing synthetic fertilizers with *Trichoderma* biofertilizer. Additionally, this study determined that CTA, with the multiple effects mentioned above, could decrease labors and costs in production.

## Materials and Methods

Healthy 2-year-old and 4-year-old *C. chinensis* plants (with rhizosphere soil) were collected from the *C. Chinensis* fields in Fengmu, Shizhu City, Chongqing, China (108°46′E, 30°25′N, altitude: 1,366 m). Soil was taken from the top 10–15 cm of sandy loam soil (organic matter 21.53 g/kg, hydrolyzable nitrogen 209.00 mg/kg, available phosphorus 55.03 mg/kg, available potassium 260.86 mg/kg, pH 5.32) at the sampling site. The fertilizer was “Nongba” compound fertilizer (nutrient content: N: 15%, P_2_O_5_: 21%, and K_2_O: 9%, diameter range: 1.85–3.24 mm, standard: GB15063-2009; Jiangsu Dikuang Compound Fertilizer Factory, Jiangsu, China).

### Culture of *Trichoderma*

*Trichoderma* species: The *Trichoderma atroviride*, *Trichoderma longibrachiatum*, *Trichoderma hamatum*, and *Trichoderma koningiopsis* were all from the laboratory-preserved species.

Culture of *Trichoderma* strains: wheat grains were soaked in water for 20 h until swollen (humidity 45%) and drained. Then, in a 500-mL canning bottle, they were mixed with the moistened wood chips (humidity 60%) at a weight ratio of grain: wood chips = 1:10, autoclaved at 121 °C and 103 kPa for 1 h, and reautoclaved at the same temperature and pressure for 1 h after 24 h. The *Trichoderma* strains were inoculated into the cooled medium and cultured in the dark at 25 °C until the mycelium and spores covered the medium (Wu et al., 2019). The spores were washed with sterile water, filtered with four layers of sterile gauze, and then diluted to a concentration of 1 × 10^8^ CFU/mL with sterile water. The spore suspensions of the four *Trichoderma* species were mixed at equal volume to make CTA.

### Effects of CTA on *C. chinensis* growth

The 2-year-old *C. chinensis* plants were rinsed with running water. Soil was sterilized at 120 °C for 3 h in oven. Plastic free-leaching pots, with a diameter of 10 cm and a total height of 10 cm, were soaked in 1‰ potassium permanganate for 1 h of disinfection, rinsed with running water, and filled with sterilized soil, 0.45 kg/pot. Then, the *C. chinensis* plants were transferred into the pots, with four plants/pot and 20 pots/treatment, using a completely randomized design. The following treatments were applied after 20 days when the *C. chinensis* plants had already adapted to the environment.

CTA: CTA was applied to the root of *C. chinensis*, 15 mL/pot.
Fer: compound fertilizer was applied to the root of *C. chinensis*, 2 g/L,15 mL/pot.
H_2_O: sterile water was applied to the root of *C. chinensis*, 15 mL/pot.

The experiments were carried out in a greenhouse under the following conditions: light 1500 Lx for 10 h/day at 20 °C, and dark 14 h/day at 15 °C (Huang & Yang, 1994). The soil was regularly watered to keep the soil moist without waterlogging. Samples were collected after 60 days. The leaves of three plants in each pot were mixed into one sample and 20 samples/treatment were prepared to determine soluble sugar, soluble protein, and chlorophyll. The remaining plants were collected, weighed, and dried at 100 °C in oven to a constant weight. The dry plants were then weighed again.

### Effects of CTA on nutrients, enzyme activity, and microbial community of rhizosphere soil

Plastic free-leaching pots, with a diameter of 20 cm and a total height of 25 cm, was hung 5 cm from the ground to avoid cross affect. They were then soaked in 1‰ potassium permanganate for 1 h of disinfection, rinsed with running water, and filled with un-sterilized soil, 3.5 kg/pot. The 4-year-old *C. chinensis* plants with rhizosphere soil were transferred into the pots, with one plant/pot, three pots/replicate, and five replicates/treatment, using a random block design. The following treatments were applied after 20 days when *C. chinensis* plants had already adapted to the environment.

CTA: CTA was applied to the root of *C. chinensis*, 25 mL/pot.
Fer: compound fertilizer was applied to the root of *C. chinensis*, 5 g/L, 25 mL/pot.
H_2_O: sterile water was applied to the root of *C. chinensis*, 25 mL/pot.

The experiments were carried out in the same greenhouse as the test above. The soil was regularly watered to keep the soil moist without waterlogging. Soil samples were collected after 60 days. The roots of the plants were taken out of the soil. The soil loosely bound to the roots was shaken off, and the soil tightly bound to the roots was collected (Liu et al., 2014). The rhizosphere soil of the three plants in each replica was mixed into one sample, and 15 such soil samples were prepared to determine the nutrients, enzyme activities, and microbial community.

### Determination methods

#### Determination of soluble sugar, soluble protein and chlorophyll in leaf

Soluble sugar: stained by anthrone and determined with automatic microplate reader.

Soluble protein: stained by Coomassie Brilliant Blue R-250 and determined using an automatic microplate reader.

Chlorophyll: extracted with acetone and determined using an automatic microplate reader (Len et al., 2020).

#### Determination of soil nutrients

Available potassium (K): using the flame photometric method.

Available phosphorus (P): using the colorimetric method.

Hydrolyzable nitrogen (N): using the alkaline hydrolysis diffusion method.

Organic matter (OM): using the potassium dichromate method.

pH: using pH meter method (Yang, Wang & Dai, 2008).

#### Determination of soil enzyme activities

The activities of soil catalase, urease, sucrase, and alkaline phosphatase were all determined using kits from Suzhou Comin Biotechnology Co., Ltd. (Suzhou, China), following the instructions and using an automatic microplate reader. The optical density of the corresponding indicators was measured at various wavelengths, and the contents were calculated using corresponding equations.

#### Determination of soil microorganism

The soil microorganism determination was carried out by Novogene Biotech Co., Ltd. (Beijing, China). The genomic DNA of the soil samples was extracted using the CTAB method. DNA concentration and purity were monitored on 1% agarose gels. According to the concentration, DNA was diluted to 1 ng/µL using sterile water and served as the template for PCR amplification of ITS1-5F fragment (fungi) and16S rDNA V3-V4 fragment (bacteria).

#### Primer sequences

ITS1-5F fragment:

ITS5-1737F: 5′-GGAAGTAAAAGTCGTAACAAGG -3′;

ITS2-2043R: 5′- GCTGCGTTCTTCATCGATGC-3′ (Lv et al., 2020).

16S rDNA V3-V4 fragment:

341F: 5′-CCTAYGGGRBGCASCAG-3′;

806R: 5′-GGACTACHVGGGTWTCTAAT-3′ (Hong et al., 2022).

PCR reaction system (30 µL): Phusion^®^ High-Fidelity PCR Master Mix (New England Biolabs, Ipswich, MA, USA) 15 µL, primer (2 µM) 3 µL, gDNA (1 ng/µL) 10 µL, and H2O 2µL.

PCR amplification conditions were as follows: initial denaturation at 98 °C for 1 min, followed by 30 cycles of denaturation at 98 °C for 10 s, annealing at 50 °C for 30 s, elongation at 72 °C for 30 s, and, finally, 72 °C for 5 min. Mixed same volume of 1× loading buffer (contained SYB green) with PCR products and operated electrophoresis on 2% agarose gel for detection. Then, PCR products was purified with Qiagen Gel Extraction Kit (Qiagen, Hilden, Germany).

Sequencing libraries were generated using TruSeq^®^ DNA PCR-Free Sample Preparation Kit (Illumina, San Diego, CA, USA) following manufacturer’s recommendations and index codes were added. The library quality was assessed on the Qubit@ 2.0 Fluorometer (Thermo Scientific, Waltham, MA, USA) and Agilent Bioanalyzer 2100 system. At last, the library was sequenced on an Illumina NovaSeq6000 platform and 250 bp paired-end reads were generated.

Paired-end reads were assigned to samples based on their unique barcode and truncated by cutting off the barcode and primer sequence, merged using FLASH (version 1.2.7, http://ccb.jhu.edu/software/FLASH/) and raw tags were obtained. Quality filtering on the raw tags were performed under specific filtering conditions to obtain the high-quality clean tags according to the QIIME (version 1.9.1, http://qiime.org/scripts/split_libraries_fastq.html) quality controlling process. The tags were compared with the Silva database (bacteria, https://www.arb-silva.de/) and Unite Database (fungi, https://unite.ut.ee/) to detect chimera sequences using UCHIME algorithm (http://www.drive5.com/usearch/manual/uchime_algo.html). Then the chimera sequences were removed to obtain the effective tags finally. Sequences analysis were performed by Uparse software (version 7.0.1001, http://drive5.com/uparse/). Sequences with ≥ 97% similarity were assigned to the same OTUs. The representative sequence for each OTU was screened for further annotation. The Silva Database (bacteria, http://www.arb-silva.de/) and Unit Database (fungi, https://unite.ut.ee/) were used based on the Mothur (bacteria) and Blast (fungi) algorithm to annotate taxonomic information. OTUs abundance information were normalized using a standard of sequence number corresponding to the sample with the least sequences.

QIIME software (version 1.9.1) was used to calculate the alpha-diversity and beta-diversity. R software (version 2.15.3) was employed to plot dilution curves and principal coordinates analysis (PCoA) plots. According to the functional annotation and abundance information of the samples in the FUNGuild (fungi) and KEGG (bacteria) database, the top ten functions of the three treatments were compared.

### Data processing

The data were recorded in Excel 2019 and analyzed using the LSD method of one-way ANOVA and Pearson Correlation in SPSS 20.0. We considered 0.01 < *P*-value < 0.05 and *P*-value < 0.01 to be indicative of a statistically significant result.

## Results

### Effects of CTA on *C. chinensis* growth

The soluble protein of the CTA and Fer treatments were significantly higher than the H_2_O treatment (*P* < 0.01). The chlorophyll a, chlorophyll b, and total chlorophyll were significantly higher after the CTA treatment than after the Fer and H_2_O treatments *(P* < 0.01) while chlorophyll a and total chlorophyll of the Fer treatment were significantly lower than the H_2_O treatment (0.01 < *P* < 0.05). Both the fresh and dry weight were significantly higher after the CTA treatment than after the H_2_O treatment (0.01 < *P* < 0.05) (Table 1).

**Table 1:** Effects of CTA on *Coptis chinensis* growth.

Treatment	Soluble sugar (mg/g fresh weight)	Soluble protein (mg/g fresh weight)	Chlorophyll a (mg/g fresh weight)	Chlorophyll b (mg/g fresh weight)	Total chlorophyll (mg/g fresh weight)	Fresh weight (g)	Dry weight (g)
CTA	109.22 ± 21.07Aa	5.1 ± 0.63Aa	2.61 ± 0.38Aa	1.14±0.15Aa	3.75 ± 0.53Aa	1.76 ± 0.27Aa	0.47 ± 0.06Aa
Fer	117.23 ± 21.09Aa	4.87 ± 0.56Aa	2 ± 0.29Bc	0.91 ± 0.11Bb	2.91 ± 0.39Bc	1.68 ± 0.27Aab	0.45 ± 0.08Aab
H_2_O	92.33 ± 15.49Aa	4.33 ± 0.61Bb	2.26 ± 0.45Bb	0.99 ± 0.18Bb	3.25 ± 0.63Bb	1.57 ± 0.28Ab	0.42 ± 0.07Ab

**Note:**

Data are reported as Mean ± SD. Different letters indicate significant differences among different treatments. Capital letters: *P* < 0.01; lower-case letters: 0.01 < *P* < 0.05. CTA, compound *Trichoderma* agent; Fer, compound fertilizer; H_2_O, sterile water.

### Effects of CTA on rhizosphere soil nutrients and enzymatic activities

Compared with H_2_O, CTA showed no significant effects on OM and available nutrients of *C. chinensis* rhizosphere soil (*P* > 0.05), while it increased the soil pH and activities of urease and catalase significantly (0.01 < *P* < 0.05 or *P* < 0.01) (Tables 2 and 3). The pH and activities of urease, catalase, and alkaline phosphatase were significantly lower after Fer than after CTA and H_2_O (0.01 < *P* < 0.05 or *P* < 0.01) (Tables 2 and 3).

**Table 2:** Effects of CTA on rhizosphere soil nutrients.

Treatment	Organic matter (g/kg)	Hydrolyzable nitrogen (mg/kg)	Available phosphorus (mg/kg)	Available potassium (mg/kg)	pH
CTA	21.36 ± 1.33Aa	152.58 ± 5.76Bb	45.40 ± 3.17Bb	256.89 ± 5.16Bb	5.13 ± 0.08Aa
Fer	21.52 ± 0.22Aa	261.43 ± 18.11Aa	50.31 ± 8.3Aa	315.33 ± 11.95Aa	4.40 ± 0.06Bc
H_2_O	21.60 ± 1.40Aa	151.81 ± 5.32Bb	44.94 ± 6.87Bb	252.86 ± 6.46Bb	5.04 ± 0.06Ab

**Note:**

Data are reported as Mean ± SD. Different letters indicate significant differences among different treatments. Capital letters: *P* < 0.01; lower-case letters: 0.01 < *P* < 0.05. CTA, compound *Trichoderma* agent; Fer, compound fertilizer; H_2_O, sterile water.

**Table 3:** Effects of CTA on rhizosphere soil enzyme activities.

Treatment	Urease [μg/(d.·g)]	Sucrase [mg/(d·.g)]	Catalase [mmol/(d.·g)]	Alkaline phosphatase [μmol/(d.·g)]
CTA	585.13 ± 45.78Aa	6.57 ± 0.30Aa	25.36 ± 0.61Aa	1.48 ± 0.29Aa
Fer	214.88 ± 14.17Cc	6.27 ± 0.57Aa	18.26 ± 1.15Bc	0.75 ± 0.14Bb
H_2_O	511.15 ± 10.83Bb	5.96 ± 0.45Aa	23.24 ± 0.65Ab	1.22 ± 0.12ABa

**Note:**

Data are reported as Mean ± SD. Different letters indicate significant differences among different treatments. Capital letters: *P* < 0.01; lower-case letters: 0.01 < *P* < 0.05. CTA, compound *Trichoderma* agent; Fer, compound fertilizer; H_2_O, sterile water.

### Effects of CTA on rhizosphere microbial communities

We obtained 1,506,050 raw tags using high-throughput sequencing of the 15 soil samples’ fungi, and 972,921 effective tags were obtained after processing, with an average efficiency of 64.60%. After clustering,19,697 OTUs were obtained. The annotated fungal OTUs of the 15 samples involved 18 phyla, 73 classes, 183 orders, 429 families, 941 genera, and 1,452 species.

We obtained1,433,061 raw tags using high-throughput sequencing of the 15 soil samples’ bacteria, and 955,153 effective tags were obtained after processing, with an average efficiency of 66.65%. After clustering, 41,597 OTUs were obtained. The annotated bacterial OTUs of the 15 samples involved 59 phyla, 147 classes, 312 orders, 417 families, 624 genera, and 300 species.

**Figure 1:**
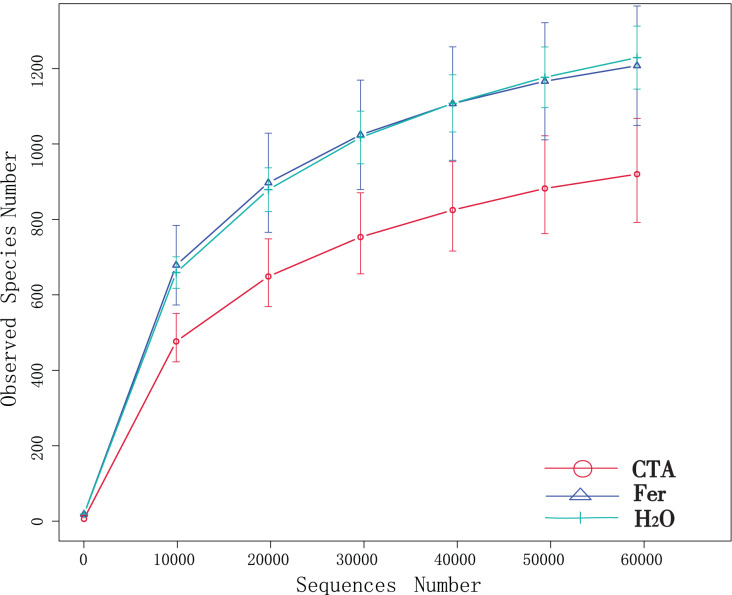
Dilution curves of rhizosphere fungi. CTA, compound *Trichoderma* agent; Fer, compound fertilizer; H_2_O, sterile water.

**Figure 2:**
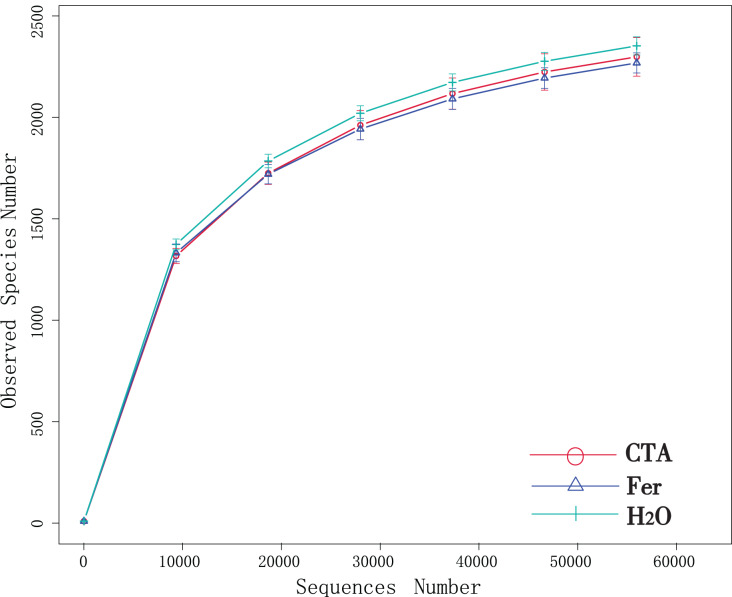
Dilution curves of rhizosphere bacteria. CTA, compound *Trichoderma* agent; Fer, compound fertilizer; H_2_O, sterile water.

Although new OTUs still appeared when the sequencing length was more than 10,000 reads, the curve was flattened, which indicated that the sampling was reasonable and the current sequencing depth was sufficient to tell the diversity of the fungal (Fig. 1) and bacterial (Fig. 2) community contained in the sample.

#### Effects of CTA on rhizosphere microbial diversity

**Figure 3:**
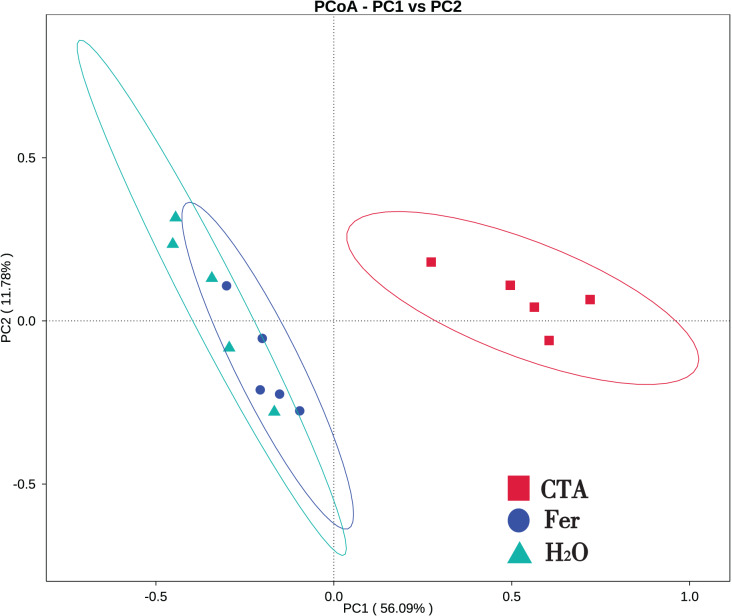
Principal coordinate analysis (PCoA) of rhizosphere fungal communities based on weighted unifrac distance. Principal coordinates analysis (PCoA) represents the differences in the rhizosphere fungal community among three treatments (CTA, Fer and H_2_O). Different colored shapes represent different groups. CTA, compound *Trichoderma* agent; Fer, compound fertilizer; H_2_O, sterile water.

**Figure 4:**
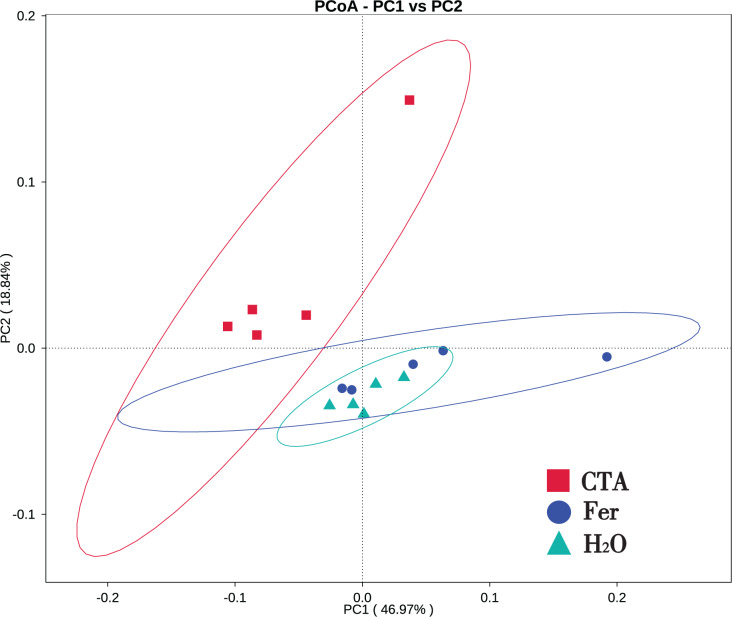
Principal coordinate analysis (PCoA) of rhizosphere bacterial communities based on weighted unifrac distance. Principal coordinates analysis (PCoA) represents the differences in the rhizosphere bacterial community among three treatments (CTA, Fer and H_2_O). Different colored shapes represent different groups. CTA, compound *Trichoderma* agent; Fer, compound fertilizer; H_2_O, sterile water.

In terms of alpha-diversity, the observed species, Shannon index, Simpson index, Chao1 index, and ACE index of fungi as well as the Shannon index and Simpson index of bacteria were significantly lower after CTA than after Fer and H_2_O (0.01 < *P* < 0.05 or *P* < 0.01), The bacteria/fungi (B/F) value was significantly higher after CTA than after Fer and H_2_O (0.01 < *P* < 0.05), while no significant difference was observed between the Fer and H_2_O (*P* > 0.05) (Table 4). With respect to the beta-diversity, the PCoA plot showed that the rhizosphere fungi and bacteria of all samples were basically divided into three groups. The PCoA plot explained 56.09% (PC1) and 11.78% (PC2) of the variation in fungal communities (Fig. 3), and 46.97% (PC1) and 18.84% (PC2) of the variation in bacterial communities (Fig. 4). The Fer and H_2_O were very close to each other on PC1 and PC2, indicating similar fungal and bacterial community structures. They were both far from CTA, indicating that both the fungal and bacterial community structures of CTA were quite different from the other two treatments (Figs. 3 and 4).

**Table 4:** Effects of CTA on alpha-diversity of rhizosphere microorganism.

	Treatment	CTA	Fer	H_2_O
Bacteria	Observed species	2,474.00 ± 111.62Aa	2,465.00 ± 59.38Aa	2,599.20 ± 127.02Aa
	Shannon index	8.62 ± 0.11Bb	8.83 ± 0.18ABa	8.89 ± 0.07Aa
	Simpson index	0.99 ± 0.00Ab	0.99 ± 0.00Aab	0.99 ± 0.00Aa
	Chao1 index	2,707.95 ± 130.84Aa	2,706.73 ± 49.43Aa	3,220.77 ± 978.90Aa
	ACE index	2,747.85 ± 133.42Aa	2,742.66 ± 45.26Aa	3,005.80 ± 400.64Aa
Fungi	Observed species	977.60 ± 166.35Ab	1,248.80 ± 188.88Aa	1259.00 ± 109.31Aa
	Shannon index	4.56 ± 0.48Bb	6.54 ± 0.55Aa	6.57 ± 0.12Aa
	Simpson index	0.81 ± 0.05Bb	0.96 ± 0.01Aa	0.97 ± 0.00Aa
	Chao1 index	1,116.93 ± 206.27Ab	1,388.65 ± 203.62Aa	1,393.60 ± 147.92Aa
	ACE index	1,155.18 ± 214.03Ab	1,407.73 ± 201.51Aab	1,433.51 ± 152.72Aa
	B/F	2.60 ± 0.49Aa	2.01 ± 0.33Ab	2.08 ± 0.23Ab

**Note:**

Data are reported as Mean ± SD. Different letters indicate significant differences among different treatments. Capital letters: *P* < 0.01; lower-case letters: 0.01 < *P* < 0.05. CTA, compound *Trichoderma* agent; Fer, compound fertilizer; H_2_O, sterile water.

**Figure 5:**
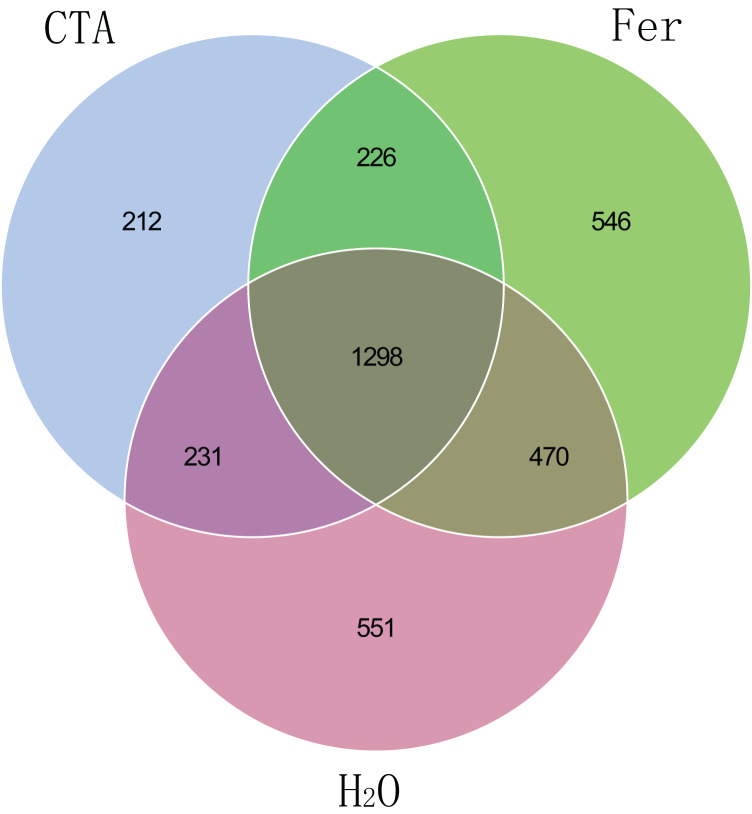
Venn diagram of rhizosphere fungal communities. Different colored shapes represent different groups. CTA, compound *Trichoderma* agent; Fer, compound fertilizer; H_2_O, sterile water.

**Figure 6:**
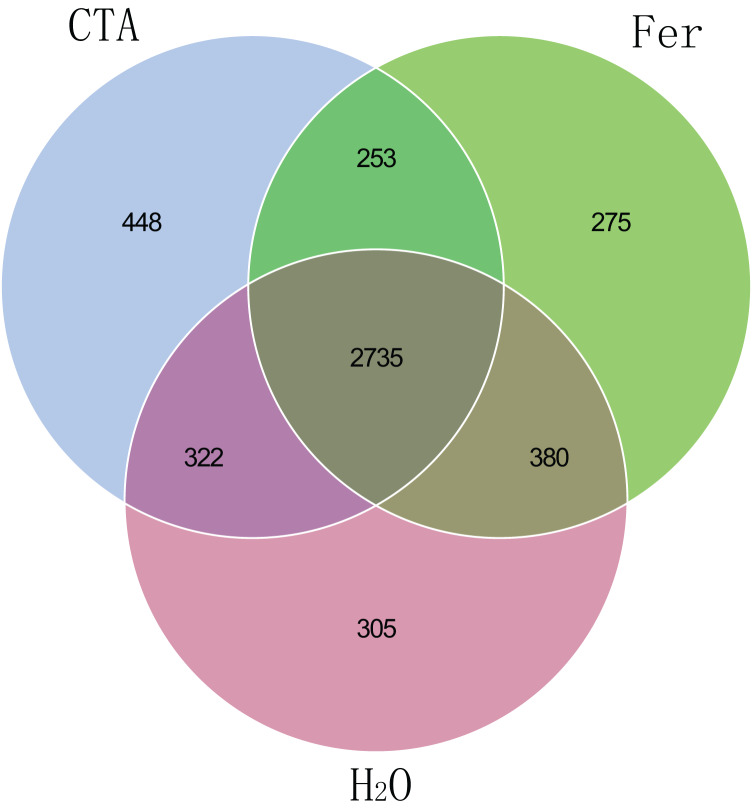
Venn diagram of rhizosphere bacterial communities. Different colored shapes represent different groups. CTA, compound *Trichoderma* agent; Fer, compound fertilizer; H_2_O, sterile water.

The fungal Venn plot showed that the three treatments had 1,298 common OTUs. There were many more unique OTUs of Fer and H_2_O than CTA (about two times as much), indicating that the fungal diversities of Fer and H_2_O were higher than that of CTA. Fer and H_2_O shared the most OTUs, and both of them shared few OTUs with CTA, indicating that the fungal community structures of Fer and H_2_O were similar to each other, but different from that of CTA. This was consistent with the aforementioned diversity analysis results (Fig. 5). The bacterial Venn plot showed that the three treatments had 2,735 common OTUs. There were fewer unique OTUs of Fer and H_2_O than CTA. Fer and H_2_O shared more OTUs, and both of them shared fewer OTUs with CTA (Fig. 6).

#### Effects of CTA on the rhizosphere microbial community structure

**Figure 7:**
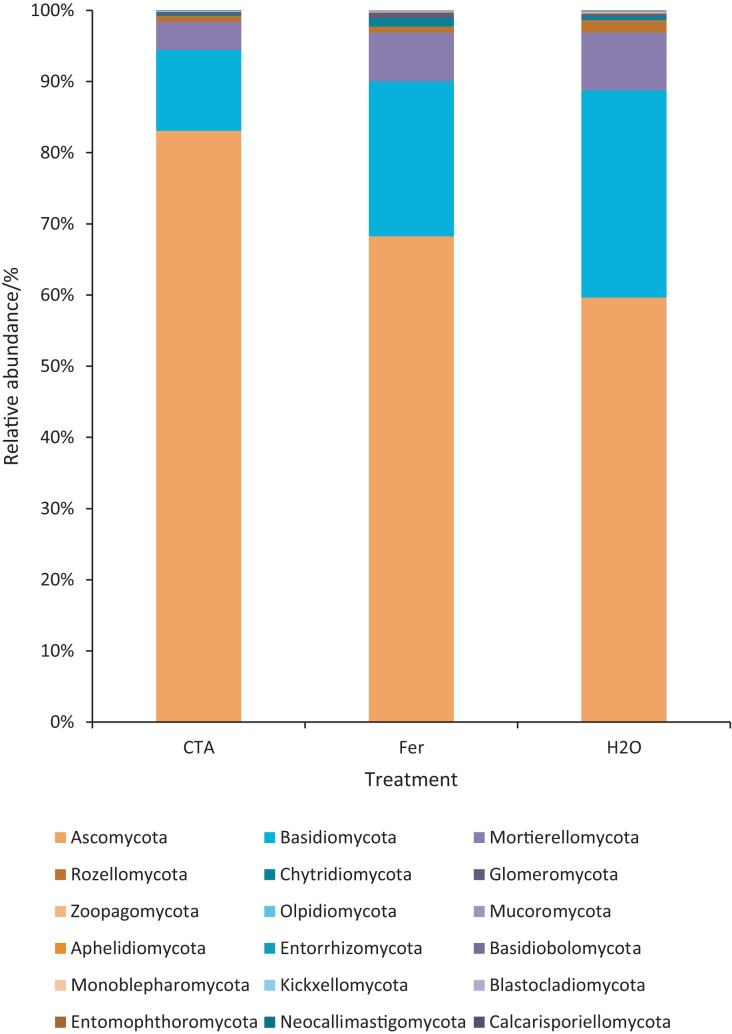
Top 20 fungi in rhizosphere soil at the phylum level. Different colored shapes represent different phyla. CTA, compound *Trichoderma* agent; Fer, compound fertilizer; H_2_O, sterile water.

**Figure 8:**
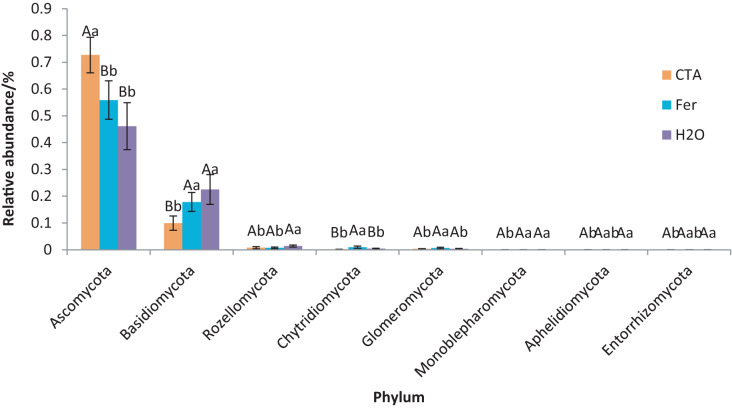
Phyla obviously different in rhizosphere fungus OTUs relative abundance within top 20 phyla. Different letters indicate significant differences among different treatments (Mean ± SD). Capital letters: *P* < 0.01; lower-case letters: 0.01 < *P* < 0.05. CTA, compound *Trichoderma* agent; Fer, compound fertilizer; H_2_O, sterile water.

The rhizosphere fungi of the three treatments were different at the phylum, genus, and species levels. Ascomycota, Mortierellomycota, Basidiomycota, *etc*. were the dominant phyla of rhizosphere fungi (Fig. 7). The relative abundance of Ascomycota was significantly higher (*P* < 0.01) while Basidiomycota was significantly lower after CTA than after H_2_O and Fer (*P* < 0.01). There were also significant differences among the relative abundance of Chytridiomycota, Glomeromycota, Rozellomycota, *etc*. in the three treatments (0.01 < *P* < 0.05 or *P* < 0.01) (Fig. 8).

**Figure 9:**
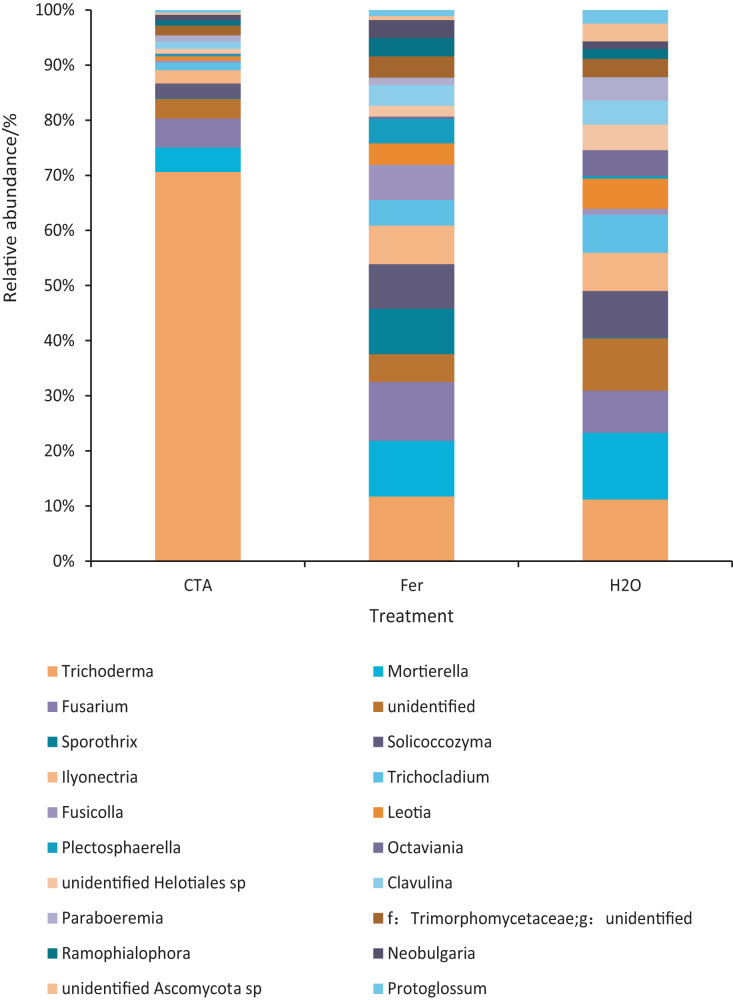
Top 20 fungi in rhizosphere soil at the genus level. Different colored shapes represent different genera. CTA, compound *Trichoderma* agent; Fer, compound fertilizer; H_2_O, sterile water.

**Figure 10:**
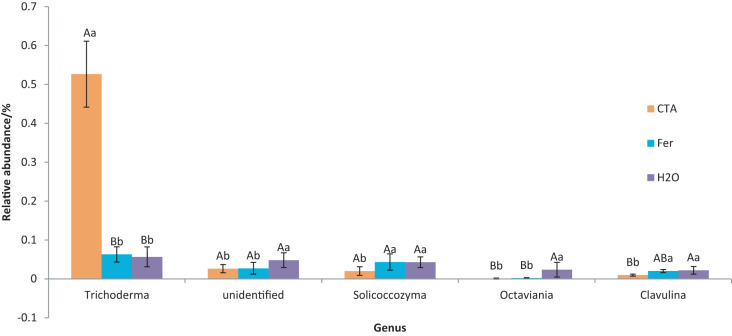
Genera obviously different in rhizosphere fungus OTUs relative abundance within top 20 genera. Different letters indicate significant differences among different treatments (Mean ± SD). Capital letters: *P* < 0.01; lower-case letters: 0.01 < *P* < 0.05. CTA, compound *Trichoderma* agent; Fer, compound fertilizer; H_2_O, sterile water.

The dominant fungal genera included *Trichoderma* sp., *Fusarium* sp., *Sporothrix* sp., *Trichocladium* sp., *etc*. (Fig. 9). The relative abundance of *Trichoderma* sp. was significantly higher after CTA than after H_2_O and Fer (*P* < 0.01). There were also significant differences among the relative abundance of *Solicoccozyma* sp., *Octaviania* sp., *Clavulina* sp., *etc*. in the three treatments (0.01 < *P* < 0.05 or *P* < 0.01) (Fig. 10).

**Figure 11:**
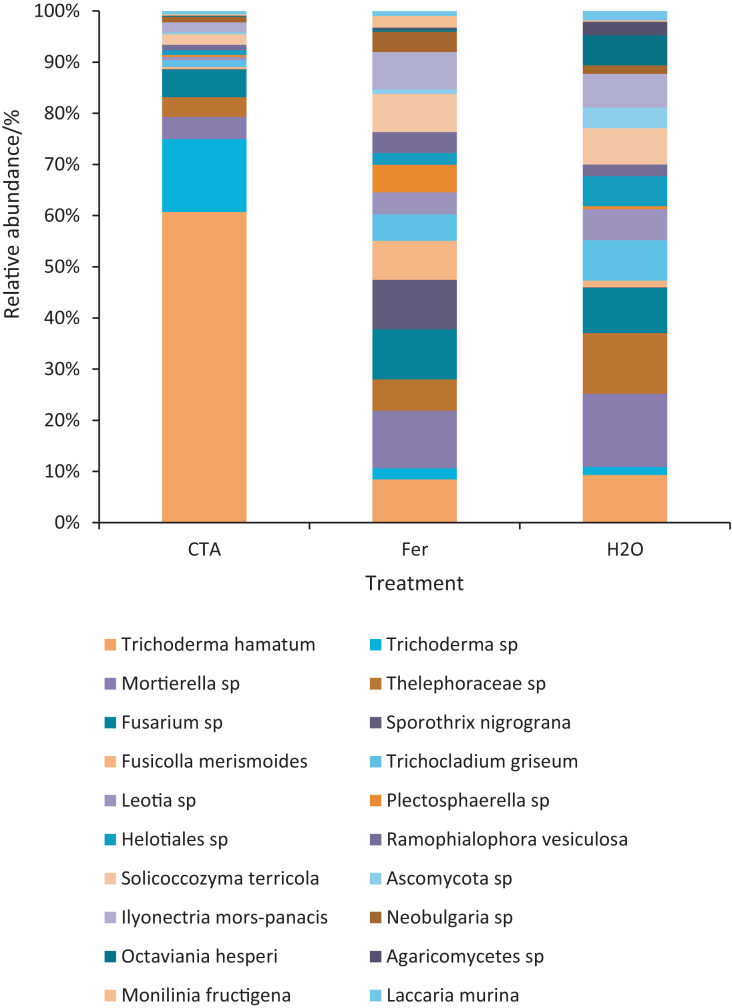
Top 20 fungi in rhizosphere soil at the species level. Different colored shapes represent different species. CTA, compound *Trichoderma* agent; Fer, compound fertilizer; H_2_O, sterile water.

**Figure 12:**
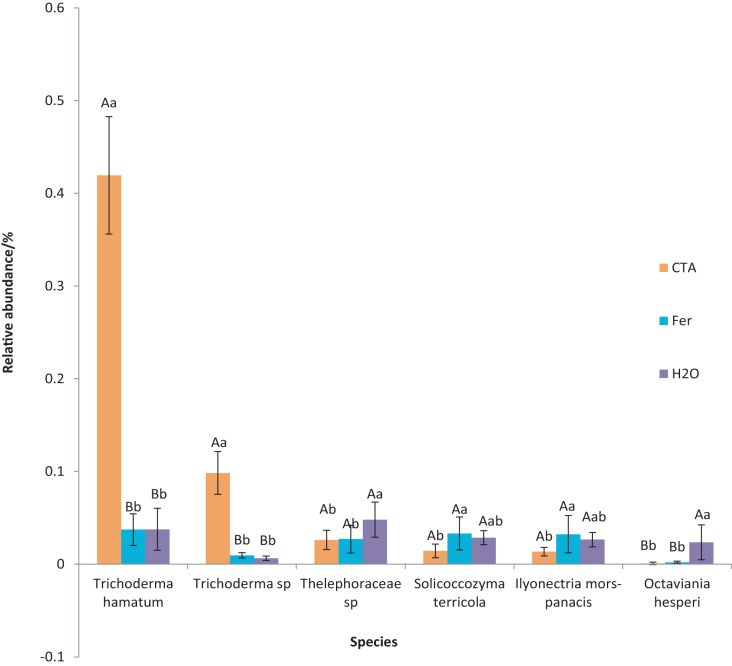
Species obviously different in rhizosphere fungus OTUs relative abundance within top 20 species. Different letters indicate significant differences among different treatments (Mean ± SD). Capital letters: *P* < 0.01; lower-case letters: 0.01 < *P* < 0.05. CTA, compound *Trichoderma* agent; Fer, compound fertilizer; H_2_O, sterile water.

The dominant fungal species were *T. hamatum*, *Sporothrix nigrograna*, *Fusicolla merismoides*, *Trichocladium griseum*, *etc*. (Fig. 11). The relative abundance of *T. hamatum* and *Trichoderma* sp. was significantly higher after CTA than after H_2_O and Fer (*P* < 0.01). The relative abundance of *Ilyonectria mors-panacis* was significantly lower after CTA than after Fer (0.01 < *P* < 0.05). There were also significant differences among the relative abundance of *Octaviania hesperi, Solicoccozyma terrea, etc*. in the three treatments (0.01 < *P* < 0.05 or *P* < 0.01) (Fig. 12).

**Figure 13:**
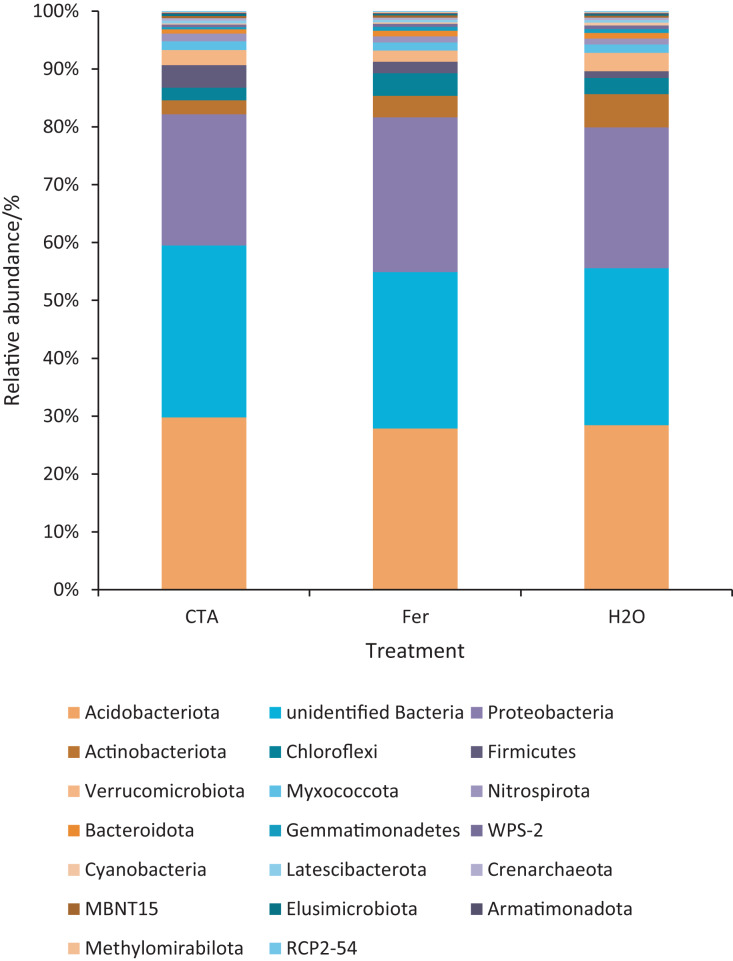
Top 20 bacteria in rhizosphere soil at the phylum level. Different colored shapes represent different phyla. CTA, compound *Trichoderma* agent; Fer, compound fertilizer; H_2_O, sterile water.

**Figure 14:**
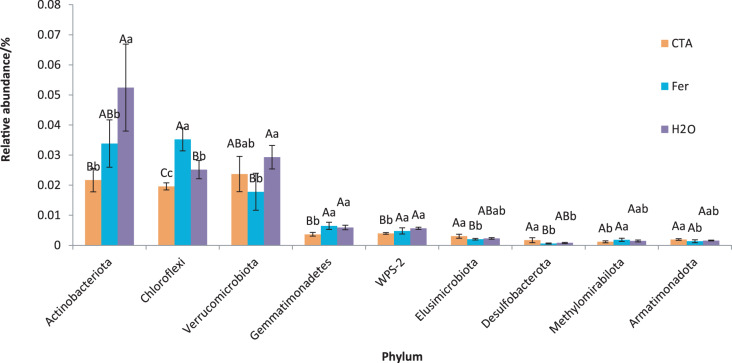
Phyla obviously different in rhizosphere bacterium OTUs relative abundance within the top 20 phyla. Different letters indicate significant differences among different treatments (Mean ± SD). Capital letters: *P* < 0.01; lower-case letters: 0.01 < *P* < 0.05. CTA, compound *Trichoderma* agent; Fer, compound fertilizer; H_2_O: sterile water.

The dominant bacterial phyla included Proteobacteria, Actinobacteriota, Firmicutes, *etc*. (Fig. 13). There were significant differences among the relative abundance of Actinobacteriota, Chloroflexi, Gemmatimonadetes, *etc*. in the three treatments (0.01 < *P* < 0.05 or *P* < 0.01) (Fig. 14).

**Figure 15:**
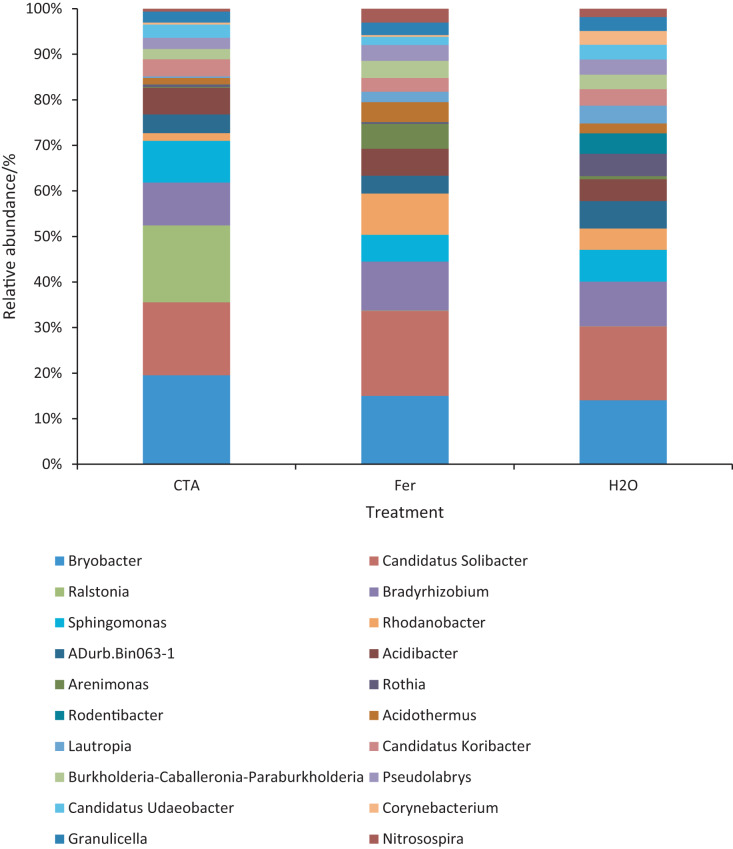
Top 20 bacteria in rhizosphere soil at the genus level. Different colored shapes represent different genera. CTA, compound *Trichoderma* agent; Fer, compound fertilizer; H_2_O: sterile water.

**Figure 16:**
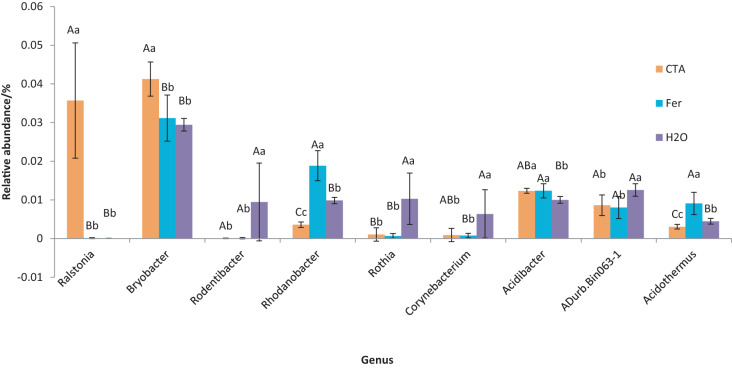
Genera obviously different in rhizosphere bacterium OTUs relative abundance within top 20 genera. Different letters indicate significant differences among different treatments (Mean ± SD). Capital letters: *P* < 0.01; lower-case letters: 0.01 < *P* < 0.05. CTA, compound *Trichoderma* agent; Fer, compound fertilizer; H_2_O, sterile water.

The dominant bacterial genera were *Bryobacter* sp., *Candidatus Solibacter* sp., *Bradyrhizobium* sp., *etc*. (Fig. 15). The relative abundance of *Corynebacterium* sp. was significantly lower after CTA than after H_2_O (0.01 < *P* < 0.05). There were also significant differences among the relative abundance of *Ralstonia* sp., *Bryobacter* sp., *Rhodanobacter* sp., *etc*. in the three treatments (0.01 < *P* < 0.05 or *P* < 0.01) (Fig. 16).

**Figure 17:**
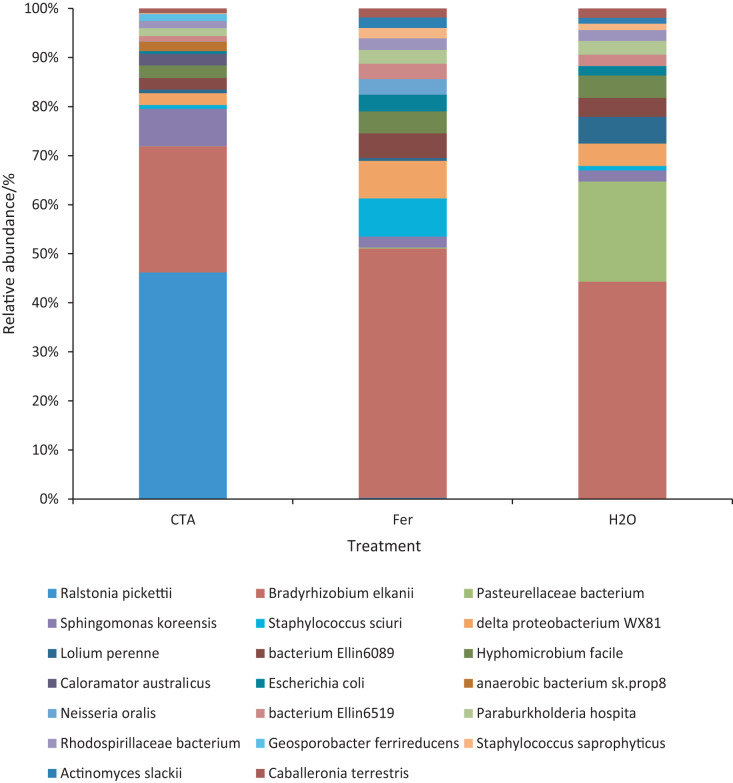
Top 20 bacteria in rhizosphere soil at the species level. Different colored shapes represent different species. CTA, compound *Trichoderma* agent; Fer, compound fertilizer; H_2_O, sterile water.

**Figure 18:**
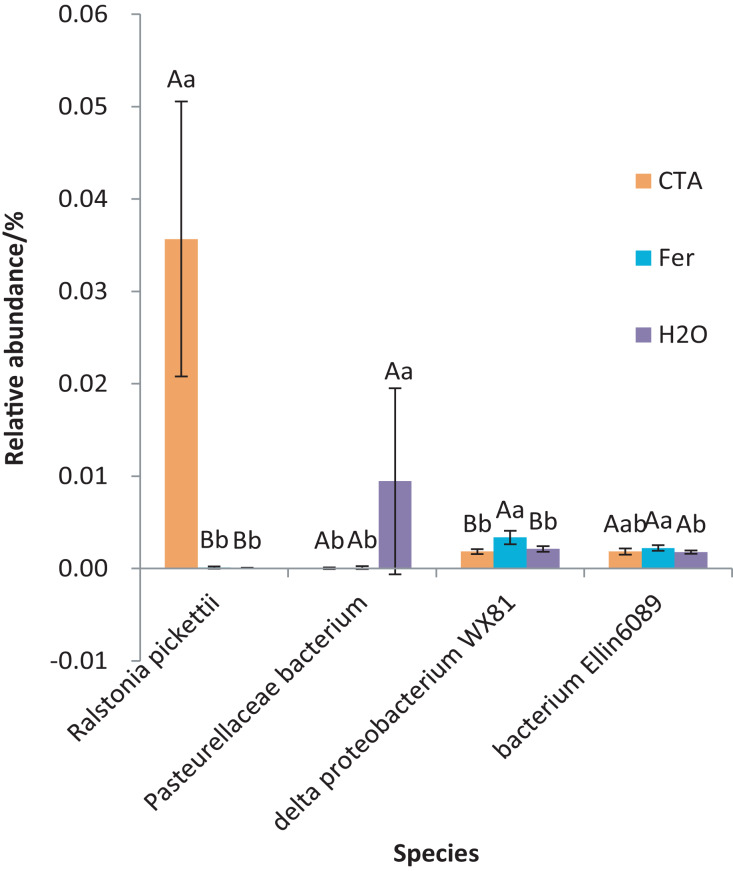
Species obviously different in rhizosphere bacterium OTUs relative abundance within top 20 species. Different letters indicate significant differences among different treatments (Mean ± SD). Capital letters: *P* < 0.01; lower-case letters: 0.01 < *P* < 0.05. CTA, compound *Trichoderma* agent; Fer, compound fertilizer; H_2_O, sterile water.

The dominant bacterial species were *Ralstonia pickettii*, *Bradyrhizobium elkanii*, Pasteurellaceae bacterium, *etc*. (Fig. 17). The relative abundance of *R. picketti* was significantly higher after CTA than after H_2_O and Fer (*P* < 0.01). There were also significant differences among the relative abundance of Pasteurellaceae bacterium, delta proteobacterium WX81, bacterium Ellin6089, *etc*.in the three treatments (0.01 < *P* < 0.05 or *P* < 0.01) (Fig. 18).

#### Function prediction of rhizosphere microorganism

The functions of the rhizosphere fungal community were divided into nine categories based on the trophic mode: Pathogen-Saprotroph-Symbiotroph, Pathotroph, Pathotroph-Saprotroph, Pathotroph-Saprotroph-Symbiotroph, Pathotroph-Symbiotroph, Saprotroph, Saprotroph-Symbiotroph, Symbiotroph, and Unassigned. Among these, Saprotroph (40.38–71.43%) and Unassigned (31.91–52.87%) were the dominant trophic modes. The relative abundance of Saprotroph was significantly higher (*P* < 0.01), while the relative abundance of Symbiotroph and Unassigned was significantly lower after CTA than after H_2_O (*P* < 0.01). The relative abundance of Unassigned was significantly lower after Fer than after H_2_O (*P* < 0.01). The relative abundance of Pathotroph-Symbiotroph, Symbiotroph, and Unassigned was significantly lower (*P* < 0.01), while the relative abundance of Saprotroph was significantly higher after CTA than after Fer (*P* < 0.01).

According to Guild, 72 functional types existed, including Plant Pathogen-Soil Saprotroph-Wood Saprotroph, Fungal Parasite-Undefined Saprotroph, Endophyte, Soil Saprotroph, *etc*. The dominant functional types were Undefined Saprotroph (39.52–70.13%), Unassigned (31.90–52.87%), Animal Pathogen-Endophyte-Plant Saprotroph-Soil Saprotroph (0.08–18.47%), and Plant Pathogen (3.90–18.22%). The relative abundance of Ectomycorrhizal was significantly lower after CTA and Fer than after H_2_O (0.01 < *P* < 0.05 or *P* < 0.01), and significantly lower after CTA than after Fer (0.01 < *P* < 0.05).

The primary functions of the rhizosphere bacterial community were divided into seven categories, including Cellular Processes, Environmental Information Processing, Genetic Information Processing, *etc*. Metabolism (48.32–48.47%) and Genetic Information Processing (19.95–20.18%) were the dominant functions. There were significant differences among the relative abundance of organismal systems, human diseases, genetic information processing, *etc*. in the three treatments (0.01 < *P* < 0.05 or *P* < 0.01).

### Correlation analysis

Correlations were found among the *C. chinensis* rhizosphere soil nutrients, enzymatic activities, and the top ten fungal and bacterial species in relative abundance. Soil alkali-hydrolysable N and available K contents were significantly negatively correlated with pH and urease, alkaline phosphatase, and catalase activities (*P* < 0.01) and were significantly positively correlated with relative abundance of *B. elkanii*, delta proteobacterium WX81, *etc*. (0.01 < *P* < 0.05 or *P* < 0.01). The activities of urease, alkaline phosphatase, and catalase were significantly positively correlated with each other (*P* < 0.01). The pH was significantly positively correlated with the activities of urease, alkaline phosphatase, and catalase (*P* < 0.01). The activities of urease, catalase, alkaline phosphatase, and pH were significantly positively correlated with the relative abundance of two *Trichoderma* fungi (0.01 < *P* < 0.05 or *P* < 0.01). The relative abundance of two *Trichoderma* fungi was significantly positively correlated with that of *R. picketti* (*P* < 0.0*1*), and significantly negatively correlated with those of delta proteobacteriumWX81 and *Mortierella* sp. (0.01 < *P* < 0.05) (Table 5).

**Table 5:** Correlation among rhizosphere soil nutrients, enzyme activities, and top 10 fungal and bacterial species.

Factor 1	Factor 2	Pearson correlation	Factor 1	Factor 2	Pearson correlation
Hydrolyzable nitrogen	Available potassium	0.979**	Catalase	Alkaline phosphatase	0.853**
Hydrolyzable nitrogen	pH	−0.954**	Catalase	Delta proteobacterium WX81	−0.781**
Hydrolyzable nitrogen	Urease	−0.937**	Catalase	*Ralstonia pickettii*	0.629*
Hydrolyzable nitrogen	Catalase	−0.898**	Catalase	*Bradyrhizobium elkanii*	−0.521*
Hydrolyzable nitrogen	Alkaline phosphatase	−0.755**	Catalase	Bacterium Ellin6089	−0.546*
Hydrolyzable nitrogen	Delta proteobacterium WX81	0.789**	Catalase	*Trichoderma hamatum*	0.688**
Hydrolyzable nitrogen	*Bradyrhizobium elkanii*	0.542*	Catalase	Trichoderma sp	0.639*
Hydrolyzable nitrogen	Bacterium Ellin6089	0.646**	Catalase	*Fusicolla merismoides*	−0.520*
Available phosphorus	*Staphylococcus sciuri*	0.730**	Alkaline phosphatase	Delta proteobacterium WX81	−0.753**
Available potassium	pH	−0.947**	Alkaline phosphatase	*Ralstonia pickettii*	0.559*
Available potassium	Urease	−0.922**	Alkaline phosphatase	*Bradyrhizobium elkanii*	−0.519*
Available potassium	Catalase	−0.870**	Alkaline phosphatase	*Trichoderma hamatum*	0.646**
Available potassium	Alkaline phosphatase	−0.778**	Alkaline phosphatase	Trichoderma sp	0.582*
Available potassium	Delta proteobacterium WX81	0.747**	Delta proteobacterium WX81	*Ralstonia pickettii*	−0.554*
Available potassium	*Bradyrhizobium elkanii*	0.536*	Delta proteobacterium WX81	Bacterium Ellin6089	0.515*
Available potassium	Bacterium Ellin6089	0.640*	Delta proteobacterium WX81	*Trichoderma hamatum*	−0.548*
Available potassium	Plectosphaerella sp	0.542*	Delta proteobacterium WX81	Sporothrix nigrograna	0.539*
pH	Urease	0.974**	*Ralstonia pickettii*	*Trichoderma hamatum*	0.865**
pH	Catalase	0.923**	*Ralstonia pickettii*	Trichoderma sp	0.847**
pH	Alkaline phosphatase	0.861**	*Bradyrhizobium elkanii*	*Sphingomonas koreensis*	−0.559*
pH	Delta proteobacterium WX81	−0.805**	*Bradyrhizobium elkanii*	Caloramator australicus	−0.564*
pH	*Trichoderma hamatum*	0.558*	*Bradyrhizobium elkanii*	Bacterium Ellin6089	0.635*
pH	Trichoderma sp	0.524*	*Bradyrhizobium elkanii*	*Fusicolla merismoides*	0.599*
Urease	Catalase	0.968**	*Sphingomonas koreensis*	Caloramator australicus	0.999**
Urease	Alkaline phosphatase	0.879**	Pasteurellaceae bacterium	Mortierella sp	0.584*
Urease	Delta proteobacterium WX81	−0.820**	Pasteurellaceae bacterium	Thelephoraceae sp	0.586*
Urease	*Ralstonia pickettii*	0.583*	*Lolium perenne*	Leotia sp	0.891**
Urease	*Bradyrhizobium elkanii*	−0.547*	*Trichoderma hamatum*	Trichoderma sp	0.988**
Urease	Bacterium Ellin6089	−0.558*	*Trichoderma hamatum*	Mortierella sp	−0.541*
Urease	*Trichoderma hamatum*	0.617*	Trichoderma sp	Mortierella sp	−0.549*
Urease	Trichoderma sp	0.565*	*Fusicolla merismoides*	Trichocladium griseum	0.659**
Sucrase	Thelephoraceae sp	−0.569*	Mortierella sp	Thelephoraceae sp	0.628*

**Notes:**

Different * indicates significant differences among different treatments.

** *P* < 0.01.

* 0.01 < *P* < 0.05.

## Discussion

### Effects of CTA on *C. chinensis* growth

Many studies have shown that *Trichoderma* fungi can promote plant growth. When *T. asperellum* and *T. harzianum* were applied to cucumber seedlings, the plant heights, stem diameters, leaf areas, whole plant fresh weights, and chlorophyll contents were all significantly higher than those of the control (Guangshu et al., 2021). *Trichoderma* fungi work by releasing auxin (López-Bucio, Pelagio-Flores & Herrera-Estrella, 2015), activating soil nutrition and enzymes (Fu et al., 2021). Soluble protein, chlorophyll, and weight are the indices of plant growth. Compared with H_2_O, CTA can increase individual weight, soluble protein, and chlorophyll content while compound fertilizer decreased chlorophyll content in leaf. CTA performed better than compound fertilizer.

### Effects of CTA on rhizosphere soil nutrients and enzyme activities

Many studies have reported on the soil improvements caused by *Trichoderma* fungi: increased enzyme activities of soil, adsorption of heavy metals, and adjustment of soil acidity (Fu et al., 2021). Soil enzymes are essential indicators of soil fertility and are involved in soil material and energy metabolism. The sources of soil enzymes mainly include plant root exudates, soil microbial exudates, animal releases, and the decomposition of animal and plant residues (Jiang et al., 2009). Urease hydrolyzes urea and promotes the transformation of N fertilizer, showing the N supply capacity of the soil (Li et al., 2018b). Catalase decomposes H_2_O_2_, which is produced during plant nutrient metabolism, into H_2_O and O_2_, thereby alleviating the toxicity of hydrogen peroxide to soil organisms (Chu, Zhang & Shi, 2015; Visser & Parkinson, 1992). Phosphatase hydrolyzes soil organic P and improves P availability (Xu et al., 2020). Alkaline phosphatase activity is also one of the main soil factors affecting the growth of *C. chinensis* (Duan et al., 2020). Soil pH affects the activities of soil enzymes, nutrient availability, plant growth, and microbial community structure, and is a crucial indicator of soil fertility (Jiang, 2018). Soil acidification lowers nutrient availability, inhibits soil enzyme activity, reduces microbial diversity, damages plant roots, and eventually compromises crop yield and quality (Liang, 2021).

The results of this study revealed that artificially added CTA could increase the activities of urease and catalase in the *C. chinensis* rhizosphere soil, while compound fertilizer decreased the activities of urease, catalase, and alkaline phosphatase, which was consistent with the previous study. After *T. hamatum* was applied to mung bean, the activities of soil urease, alkaline phosphatase, catalase, and sucrase were all elevated (Baazeem et al., 2021). The activities of sucrase, acid phosphatase, urease, and catalase in the rhizosphere soil of *Pinus sylvestris* seedlings applied with *Trichoderma harzianum* and *Trichoderma virens* were all significantly higher than those in the control (Halifu et al., 2019), while the activities of sucrase, urease, and alkaline phosphatase in the rhizosphere soil of pepper applied with synthetic fertilizer alone were lower than those in the soil treated with *Trichoderma* organic fertilizer, partially replacing synthetic fertilizer (Jin et al., 2021).Therefore, *Trichoderm*a increased the enzyme activities of the soil, while synthetic fertilizer inhibited them. *Trichoderma* spp. enlarged the contact area between the root and the soil and boosted the secretion of extracellular enzymes such as sucrase and urease, so as to improve the activities of soil enzymes (Halifu et al., 2019). In addition, *Trichoderma* elevated the enzyme activities by increasing soil pH, while the compound fertilizer decreased pH, possibly due to the induced soil acidification that inhibited the enzyme activities (Liang, 2021).

CTA significantly increased soil pH. The application of *T. harzianum* to *Brassica campestris* altered the soil pH from 6.75 to 6.97 (Liu et al., 2020), while *Trichoderma* reduced pH in saline-alkali soil (Jian et al., 2021). This indicated that *Trichoderma* had a two-way regulating effect on soil pH. Presumably, application of a large amount of *Trichoderma* to the soil affected the balance of the soil microbial communities, which affected the root exudates and thus changed the soil pH (Wang, 2014). However, compound fertilizer increased soil acidity. Numerous other studies have also shown that the long-term application of synthetic fertilizers, especially N fertilizers, led to soil acidification (Tian & Niu, 2015). The soil pH in most *C. chinensis*-producing areas was acidic (Chen et al., 2005), and the ideal soil pH for *C. chinensis* was 5.5–7.0 (Sun, 2006). Therefore, compared with compound fertilizer, CTA performed better at adjusting the soil pH to the ideal range for *C. chinensis*.

CTA could not improve soil nutrient and OM content. However, the combination of CTA and organic fertilizers could overcome this drawback. When *T. longibrachiatum* was applied to mangoes in combination with organic fertilizer, the yield increased by 13% compared to using no fertilizer, 7% compared with synthetic fertilizer applied alone, and 6% compared with organic fertilizer applied alone (Zhu et al., 2021).

### Effects of CTA on soil microbial community structure and function

Microorganisms are an important part of the soil and play a critical role in the decomposition of OM and nutrient cycling (Jiao et al., 2019). They also affect the physicochemical properties of the soil (Sharma et al., 2017). The diversity of soil microorganisms affects soil fertility (Tahat et al., 2020).

In this study, both the number and alpha-diversity of rhizosphere fungi were lower after CTA than after the other two treatments. It was also found that the alpha-diversity and number of rhizosphere fungi of *P. sylvestris* seedlings treated with *T. harzianum* and *T. virens* were lower than those of the control (Halifu et al., 2019). The alpha-diversity of bacteria was lower after CTA than after the other two treatments. Yu et al. (2020) found that, after *Trichoderma brevicompactum* treatment, the alpha-diversity and number of rhizosphere bacteria were lower than those of the control. This demonstrated the inhibitory effect of *Trichoderma* on other microorganisms, which is related to its biocontrol function. *Trichoderma* spp. grow fast, can occupy the growing space, and absorb the required nutrients quickly. At the same time, *Trichoderma* spp. may produce cell wall-degrading enzymes to break down microbial cells in the soil environment to absorb nutrients (Zhang, 2015).

CTA increased rhizosphere soil B/F value. It was also found that *Trichoderma* bio-organic fertilizer increased rhizosphere soil B/F value (Pu, 2019). Soil B/F value is usually closely related to soil-borne diseases and continuous cropping obstacles. Continuous cropping tends to increase fungi and decrease bacteria in soil, which causes soil-borne diseases. For example, after continuous cropping of cucumber, the soil turns from a bacterial type to fungal type (Du et al., 2017). It was concluded that bacterial-type soil was an indicator of higher fertility and vitality, while the fungal-type soil was a sign of soil failure (Feng et al., 1999). Therefore, CTA improved the soil microbial community.

CTA significantly altered the microbial community structure in the soil at the phylum, genus, and species levels. In this study, the relative abundance of Ascomycota was significantly higher, and the relative abundance of Basidiomycota was significantly lower after CTA than after H_2_O and Fer. It was revealed that healthy soil contained a higher abundance of Ascomycota, while sub-healthy soil had more abundant Basidiomycota (Qiao, 2021), indicating that CTA-treated soil was healthier than H_2_O-or Fer-treated soil. CTA decreased the abundance of harmful *Corynebacterium* sp. (Guo et al., 2002), and *I. mors-panacis* (Farh, Kim & Singh, 2017) in the soil. *Ilyonectria* sp. is comprised of various phytopathogenic fungi (Bischoff Nunes & Goodwin, 2022), including the pathogen responsible for the root rot disease of *C. chinensis* (Wu et al., 2021). A large number of studies showed that *Trichoderma* could reduce the number of various phytopathogenic microorganisms in the soil, thereby controlling the disease. When *Trichoderma Gamsii* and *T. harzianum* were tested against *Fusarium Pseudograminearum*, a significant decrease in crown rot symptoms was achieved in wheat, with reduced pathogen populations in soil (Stummer et al., 2022). CTA increased the abundance of *Trichoderma* spp. and *R. picketti* which could degrade phenolic acid, an allelopathic compound in the soil, that can control continuous cropping obstacles of C. *chinensis*.

The *Tichoderma* spp. were still dominant in *C. chinensis* rhizosphere soil 60 days after application of CTA, showing a long lifetime. Other studies also revealed that *Tichoderma* can exist in soil for a long time. The total valid term of rhizosphere *Tichoderma atroviride* applied to loquat was 75 days (Lu et al., 2020). When *Trichoderma asperellum* was applied to cucumber, its content in rhizosphere soil reached its peak at 70 d (Huo et al., 2016).

There were differences among the microbial functions of the three treatments. CTA decreased the abundance of Pathotroph, which showed that CTA inhibited some pathogenic fungi, and increased the abundance of Saprotroph, due to the fact that *Trichoderma* are Saprophytic fungi.

### Correlation among rhizosphere soil nutrients, enzyme activities, and microbial community structure

An significant positive correlation was found between pH and urease, alkaline phosphatase, and catalase activities. Acosta-Martínez & Tabatabai (2000) also found that the activities of urease, amidase, alkaline phosphatase, and phosphodiesterase were significantly positively correlated with soil pH, which indicated that the increase in soil pH within a certain range benefited the soil enzymes, while soil acidification inhibited enzyme activity (Zhao et al., 2022). This was due to the pH which directly affected the speed of soil enzymes participating in biochemical reactions. When the pH was beyond the ideal range, it inhibited enzyme activities (Zhao, 2011). At the same time, the activities of urease, catalase, and alkaline phosphatase, as well as pH, were all significantly positively correlated with the abundance of two *Trichoderma* fungi, indicating that the application of CTA elevated pH and enzyme activities.

## Conclusion

CTA increased soluble protein, chlorophyll, and individual weight of *C. chinensis* plants while compound fertilizer reduced chlorophyll. CTA increased the activities of soil enzymes and pH in the *C. chinensis* rhizosphere soil, whereas the compound fertilizer reduced them. CTA showed no significant effects on soil nutrients and organic matter, while it decreased the fungal number and alpha-diversity of fungi and bacteria and increased B/F value, which improved the rhizosphere microbial community. Both CTA and the compound fertilizer significantly altered the soil microbial community structure. CTA improved soil quality by increasing beneficial Ascomycota and *R. picketti* and decreasing harmful Basidiomycota, *I. mors-panacis*, and *Corynebacterium* sp.

In summary, synthetic fertilizers damage soil fertility and their overuse might be associated with the occurrence of root rot disease. CTA can promote *C. chinensis* growth, improve soil, and decrease the incidence and severity of *C. chinensis* root rot disease, which makes it possible to replace synthetic fertilizers, at least partially, with CTA as biofertilizer in *C. chinensis* production. Combining it with organic fertilizer will increase the potential of *Trichoderma*. Previous studies on *C. chinensis* root rot control mainly focused on synthetic pesticides, which have been proved ineffective in *C. chinensis* production. Replacement of synthetic fertilizers with a compound *Trichoderma* agent which serves as both a biopesticide and biofertilizer provides a new solution.

## Supplemental Information

10.7717/peerj.15652/supp-1 Supplemental Information 1 Effects of CTA on *Coptis chinensis* growth. Raw data for Table 1.

10.7717/peerj.15652/supp-2 Supplemental Information 2 Effects of CTA on rhizosphere soil nutrients. Raw data for Table 2.

10.7717/peerj.15652/supp-3 Supplemental Information 3 Effects of CTA on rhizosphere soil enzyme activities. Raw data for Table 3.

10.7717/peerj.15652/supp-4 Supplemental Information 4 Effects of CTA on alpha-diversity of rhizosphere microorganism. Raw data for Table 4.

10.7717/peerj.15652/supp-5 Supplemental Information 5 Relative abundance of top 10 fungal and bacterial species. Raw data for Table 5.

10.7717/peerj.15652/supp-6 Supplemental Information 6 Raw data of bacterial function prediction.

10.7717/peerj.15652/supp-7 Supplemental Information 7 Raw data of fungal function prediction according to Guild.

10.7717/peerj.15652/supp-8 Supplemental Information 8 Relative abundance of different fungal trophic mode. Raw data of fungal function prediction according to Mode.

10.7717/peerj.15652/supp-9 Supplemental Information 9 Microbial community structure at the time of CTA application (on 20 days post sowing).

## Additional Information and Declarations

### Data Availability

The following information was supplied regarding data availability: The data is available at NCBI SRA: SAMN27533081 to SAMN27533110; PRJNA820511.

## References

[ref-1] Acosta-Martínez V, Tabatabai MA (2000). Enzyme activities in a limed agricultural soil. Biology & Fertility of Soils.

[ref-3] Baazeem A, Almanea A, Manikandan P, Alorabi M, Vijayaraghavan P, Abdel-Hadi A (2021). In vitro antibacterial, antifungal, nematocidal and growth promoting activities of *Trichoderma hamatum* FB10 and its secondary metabolites. Journal of Fungi.

[ref-4] Bian ZL (2022). Preparation and evaluation of an engineered *Pseudomonas* as microbial inoculation in field trials.

[ref-5] Bischoff Nunes I, Goodwin PH (2022). Interaction of ginseng with *Ilyonectria* root rot pathogens. Plants.

[ref-6] Chen SJ, Zhong GY, Zhang H, Wang ZY (2005). Preliminary studies on the nutrient characters of different kinds of Chinese goldthread soil. China Journal of Chinese Materia Medica.

[ref-7] Chu SZ, Zhang NM, Shi J (2015). Research on the variation trend of greenhouse soil hydrogen peroxidase activities in Yunnan Province. Chinese Agricultural Science Bulletin.

[ref-8] Cohen SP, Leach JE (2020). High temperature-induced plant disease susceptibility: more than the sum of its parts. Current Opinion in Plant Biology.

[ref-9] Du L, Huang BJ, Du NS, Guo SR, Shu S, Sun J (2017). Effects of garlic/cucumber relay intercropping on soil enzyme activities and the microbial environment in continuous cropping. HortScience.

[ref-10] Duan YY, LIiu XH, Wu JQ, Zhou WX, Guo XL, You JM, Tang T, Wang FF, Guo J (2020). Effects of intercropping patterns on physiological and growth traits of *Coptis chinensis* and rhizospheric soil physicochemical properties. Chinese Journal of Ecology.

[ref-11] Duffy BK, Défago G (1999). Macro- and microelement fertilizers influence the severity of fusarium crown and root rot of tomato in a soilless production system. Hortscience A Publication of the American Society for Horticultural Science.

[ref-12] Farh EA, Kim YJ, Singh P (2017). Cross interaction between *Ilyonectria mors-panacis* isolates infecting Korean ginseng and ginseng saponins in correlation with their pathogenicity. Phytopathology.

[ref-13] Feng HS, Wan SB, Zuo XQ, Cheng B (1999). Rhizosphere soil main microbial taxa variation and its correlation with yield in continuous cropping of Peanut. Peanut Science and Technology.

[ref-14] Fu J, Xiao Y, Wang YF, Liu ZH, Yang KJ (2021). Saline-alkaline stress in growing maize seedlings is alleviated by *Trichoderma asperellum* through regulation of the soil environment. Scientific Reports.

[ref-15] Gai XH, Liu SX, Ren T, Liu Y, Tian CW (2018). Research progress on chemical constituents of Coptidis Rhizoma and its pharmacological activities. Chinese Traditional and Herbal Drugs.

[ref-16] Gherbawy YA, Hussein NA, Al-Qurashi AA (2014). Molecular characterization of *Trichoderma* populations isolated from soil of Taif City, Saudi Arabia. International Journal of Current Microbiology and Applied Sciences.

[ref-17] Guangshu M, Mei L, Mingxin L, Yurong C, Hua L (2021). Effect of *Trichoderma* on cucumber damping-off and physiological characteristics. Chinese Journal of Biological Control.

[ref-18] Guo JH, Cai YJ, Chen YY, Guo YY (2002). Taxonomy of plant pathogenic coryneform bacteria. Microbiology.

[ref-19] Halifu S, Deng X, Song XH, Song RQ (2019). Effects of two *Trichoderma* strains on plant growth, rhizosphere soil nutrients, and fungal community of *Pinus sylvestris* var. mongolica annual seedlings. Forests.

[ref-20] Hong ZF, Zhang NC, Deng A, Qiu RL, Lin QQ, Yao YQ (2022). Bacterial diversity and community composition in the *Phragmites australis* rhizosphere by cometabolism. China Environmental Science.

[ref-21] Hou MY, Zhang L, Wang ZW, Yang DL, Wang LL, Xiu WM, Zhao JN (2017). Estimation of fertilizer usage from main crops in China. Journal of Agricultural Resources and Environment.

[ref-22] Huang ZF, Yang MQ (1994). Biological characteristics of coptis and the major cultivation techniques for it. Journal of Southwest Agricultural University.

[ref-23] Huo ZD, Song SQ, Gao YF, Shi YX, Li BJ (2016). Detection of *Trichoderma asperellum* colonization in soils by real-time fluorescent quantitative PCR. Journal of Plant Protection.

[ref-24] Ji S, Liu Z, Liu B, Wang Y, Wang J (2020). The effect of *Trichoderma* biofertilizer on the quality of flowering Chinese cabbage and the soil environment. Scientia Horticulturae.

[ref-25] Jian F, Yao X, Wang YF, Liu ZH, Yang KJ (2021). *Trichoderma asperellum* alters fungal community composition in saline-alkaline soil maize rhizospheres. Soil Science Society of America Journal.

[ref-26] Jiang XY (2018). Analysis on the spatial and temporal variations of soil pH and its influencing factors in the core area of Chengdu plain.

[ref-27] Jiang JP, Xiong YC, Jiang HM, Ye DY, Song YJ, Li FM (2009). Soil microbial activity during secondary vegetation succession in semiarid abandoned lands of loess plateau. Pedosphere.

[ref-28] Jiao S, Xu YQ, Zhang J, Hao X, Lu YH (2019). Core microbiota in agricultural soils and their potential associations with nutrient cycling. mSystems.

[ref-29] Jin YZ, Xiong YN, Sun X, Li JY, Li X, He SP, Li CX (2021). Effects of combined application of chemical fertilizer reduction with *Trichoderma* organic fertilizer on yield and quality of pepper and enzyme activities in rhizosphere soil. Journal of Sichuan Agricultural University.

[ref-31] Len F, Yang ZJ, Wu YC, He DW (2020). Physiological and photosynthetic characteristics and active component contents of *Polygonum multiflorum* Thunb under different soil pH values. Acta Botanica Boreali-Occidentalia Sinica.

[ref-32] Li YT, Hwang SG, Huang YM, Huang CH (2018a). Effects of *Trichoderma asperellum* on nutrient uptake and *Fusarium wilt* of tomato. Crop Protection.

[ref-33] Li JH, Xia Q, Yang ZP, Zhang X, Sun M, Gao ZQ, Yang ML (2018b). Effects of different kinds of basal application slow-release fertilizers and dosages on vertical distribution of urease activity in wheat rhizosphere soil. Journal of Shanxi Agricultural Sciences.

[ref-34] Liang JW (2021). Effects of quicklime amounts on acidic soil and pre-growth of tobacco.

[ref-35] Liu ZY, Wang RF, Qiao CC, Zhang N, Shen ZZ, Li R, Shen QR (2020). Effects of *Trichoderma* bio-organic fertilizer application on yield and soil microflora in Chinese cabbage and cabbage rotation system. Journal of Nanjing Agricultural University.

[ref-36] Liu FC, Xing SJ, Ma HL, Du ZY, Ma BY (2014). Effects of inoculating plant growth-promoting rhizobacteria on the biological characteristics of walnut (*Juglans regia*) rhizosphere soil under drought condition. Chinese Journal of Applied Ecology.

[ref-39] López-Bucio J, Pelagio-Flores R, Herrera-Estrella A (2015). Trichoderma as biostimulant: exploiting the multilevel properties of a plant beneficial fungus. Scientia Horticulturae.

[ref-37] Lu HJ, Xie XY, Tao HZ, Shen YM, Wang D (2020). Determination of colonization ability of endophytic *Trichoderma* P3.9 strain in loquat rhizosphere soil. Jiangsu Agricultural Sciences.

[ref-38] Lv BB, Zhang GY, Zhang LP, Liu Z, Fan QL, Yao Z (2020). Effects of rape rhizosphere and biofumigation on the community of continuous cropping soil fungi. Northern Horticulture.

[ref-40] Massah J, Azadegan B (2016). Effect of chemical fertilizers on soil compaction and degradation. Agricultural Mechanization in Asia, Africa and Latin America.

[ref-41] Niño-Savala AJ, Zhuang Z, Ma X, Fangmeier A, Li HF, Tang AH, Liu XJ (2019). Cadmium pollution from phosphate fertilizers in arable soils and crops: an overview. Frontiers of Agricultural Science and Engineering.

[ref-42] Pu XW (2019). Effects of *Trichoderma* bio-organic fertilizer on the growth and soil microflora of sand-pressed watermelon.

[ref-43] Qiao YY (2021). Screening of microbial indexes for soil health assessment in wheat area of Zhejiang Province.

[ref-44] Sharma A, Saha TN, Arora A, Shah R, Nain L (2017). Efficient microorganism compost benefits plant growth and improves soil health in Calendula and Marigold. Horticultural Plant Journal.

[ref-45] Sharma P, Sharma M, Raja M, Singh DV, Srivastava M (2016). Use of *Trichoderma* spp. in biodegradation of Carbendazim. The Indian Journal of Agricultural Sciences.

[ref-46] Shen G, Zhang S, Liu X, Jiang Q, Ding W (2018). Soil acidification amendments change the rhizosphere bacterial community of tobacco in a bacterial wilt affected field. Applied Microbiology and Biotechnology.

[ref-47] Stummer BE, Zhang XJ, Yang HT, Harvey PR (2022). Co-inoculation of *Trichoderma gamsii* A5MH and *Trichoderma harzianum* Tr906 in wheat suppresses in planta abundance of the crown rot pathogen *Fusarium pseudograminearum* and impacts the rhizosphere soil fungal microbiome. Biological Control.

[ref-48] Sun YF (2006). Study about effects of temperature stress on physiological and biochemical character of *Coptis chinensis* Franch.

[ref-49] Tahat MM, Alananbeh KM, Othman YA, Leskovar DI (2020). Soil health and sustainable agriculture. Sustainability.

[ref-50] Tang X, Liang F, Xu MG, Wen SL, Cai ZJ, Song FF, Gao Q (2020). A meta-analysis of effects of long-term application of chemical fertilizer on pH of farmland soil. Journal of Jilin Agricultural University.

[ref-51] Tansengco M, Tejano J, Coronado F, Gacho C, Barcelo J (2018). Heavy metal tolerance and removal capacity of *Trichoderma* species isolated from mine tailings in Itogon, Benguet. Environment and Natural Resources Journal.

[ref-52] Tian DH, Niu SL (2015). A global analysis of soil acidification caused by nitrogen addition. Environmental Research Letters.

[ref-53] Visser S, Parkinson D (1992). Soil biological criteria as indicators of soil quality: soil microorganisms. American Journal of Alternative Agriculture.

[ref-54] Wang H (2014). Study on the growth characteristic of tissue-cultured *Populus davidiana* X *P. bolleana* seedlings transplanted into fields and analysis on soil nutrient inducing by *Trichoderma aspereullm*.

[ref-55] Win TT, Bo B, Malec P, Khan S, Fu PC (2021). Newly isolated strain of *Trichoderma asperellum* from disease suppressive soil is a potential bio-control agent to suppress *Fusarium* soil borne fungal phytopathogens. Journal of Plant Pathology.

[ref-56] Wu XL, Chen DX, Liu F, Wang Y, Li LY (2021). Identification of *Coptis chinensis* root rot disease pathogenic fungi belonging to *Ilyonectria* spp. Southwest China Journal of Agricultural Sciences.

[ref-57] Wu XL, Tan J, Cui GL, Liu F, Li LY (2019). Promoting effects of *Trichoderma* atroviride on *Artemisia* annual Growth. Journal of Southwest University (Natural Science).

[ref-58] Wu XL, Wang Y, Liu F, Chen DX, Li LY (2020). Identification of *Coptis chinensis* root rot disease pathogenic *Fusarium* spp. fungi. China Journal of Chinese Materia Medica.

[ref-59] Xu CH, Lu MX, Fan DW, Chen H, Han JG (2020). Effects of wetland reclamation on kinetic characteristics of soil alkaline phosphatase. Journal of Zhejiang A & F University.

[ref-60] Yang JH, Wang CL, Dai HL (2008). Soil agricultural chemistry analysis and environmental monitoring.

[ref-61] Yu K, Li W, Liu Y, Dong A, Liu W (2020). Effects of different treatments on soil bacterial diversity. Forest Engineering.

[ref-62] Yu Z, Wang Z, Zhang Y, Wang Y, Liu Z (2021). Biocontrol and growth-promoting effect of *Trichoderma asperellum* TaspHu1 isolate from *Juglans mandshurica* rhizosphere soil. Microbiological Research.

[ref-63] Zhang FG (2015). The effects and mechanisms of putative *Trichoredma harzianum* mutant T-E5 and ITS bio-organic fertilizer on growth of cucumber.

[ref-64] Zhao J (2011). Effects of soil acidification on available soil nutrients, soil enzyme activity and characters of Whang Keumbae in pear orchards.

[ref-65] Zhao KG, Zhou ZH, Jin Y, Wang CK (2022). Effects of long-term nitrogen addition on soil carbon nitrogen, phosphorus and extracellular enzymes in *Larix gmelinii* and *Fraxinus mandshurica* plantation. Journal of Nanjing Forestry University (Natural Sciences Edition).

[ref-66] Zhu JX, Shang MN, Li R, Liu HJ, Qiao CC, Tao CY, Shen ZZ, Shen QR (2021). Development and application of specific bio-organic fertilizer containing *Trichoderma* strain for mango orchard in tropic areas. Soil and Fertilizer Sciences in China.

