# Supplementary material for: Effects of a compound Trichoderma agent on Coptis chinensis growth, nutrients, enzyme activity, and microbial community of rhizosphere soil
Source: PeerJ. 2023 Jul 12;11:e15652. doi: 10.7717/peerj.15652 (PMC10349559; doi:10.7717/peerj.15652)
Supplement: Supplemental Information 1 — Raw data for Table 1. [file peerj-11-15652-s001.docx]

| Treatment | Soluble sugar （mg/g fresh weight） | Soluble protein (mg/g fresh weight) | Chlorophyll a (mg/g fresh weight) | Chlorophyll b (mg/g fresh weight) | Total chlorophyll (mg/g fresh weight) | Fresh weight（g） | Dry weight（g） |
| --- | --- | --- | --- | --- | --- | --- | --- |
| CTA | 94.85597 | 4.318439 | 2.663096 | 1.192606 | 3.855702 | 1.628 | 0.434 |
|  | 84.8953 | 4.525847 | 2.786158 | 1.234707 | 4.020866 | 1.49 | 0.454 |
|  | 85.21391 | 4.123324 | 2.7628 | 1.194245 | 3.957045 | 2.02 | 0.501 |
|  | 118.8666 | 5.428919 | 2.790152 | 1.225047 | 4.015198 | 1.965 | 0.537 |
|  | 120.0476 | 4.983574 | 2.957216 | 1.242351 | 4.199567 | 1.654 | 0.578 |
|  | 115.9694 | 5.282294 | 3.31914 | 1.427268 | 4.746408 | 1.836 | 0.486 |
|  | 125.2864 | 5.56642 | 2.421899 | 1.077947 | 3.499847 | 1.221 | 0.392 |
|  | 119.0032 | 4.063451 | 1.846783 | 0.825409 | 2.672192 | 1.745 | 0.401 |
|  | 116.7035 | 4.745414 | 2.348757 | 1.028092 | 3.376849 | 1.975 | 0.486 |
|  | 131.1956 | 5.715401 | 2.238937 | 1.009483 | 3.248421 | 1.849 | 0.492 |
|  | 111.6448 | 4.552632 | 2.719477 | 1.172493 | 3.89197 | 2.085 | 0.445 |
|  | 135.9324 | 5.906903 | 2.73642 | 1.218941 | 3.955361 | 1.259 | 0.428 |
|  | 114.1264 | 5.927861 | 3.030367 | 1.337447 | 4.367814 | 1.957 | 0.529 |
|  | 57.00333 | 5.796116 | 2.288391 | 1.021774 | 3.310165 | 1.793 | 0.482 |
|  | 78.77695 | 4.598805 | 3.014879 | 1.27054 | 4.285419 | 1.808 | 0.552 |
|  | 89.65464 | 5.41702 | 2.847324 | 1.205189 | 4.052513 | 1.779 | 0.457 |
|  | 121.539 | 5.781614 | 2.871517 | 1.230691 | 4.102208 | 1.838 | 0.365 |
|  | 130.3617 | 5.70623 | 2.156556 | 0.967539 | 3.124095 | 2.052 | 0.561 |
|  | 131.1235 | 4.617337 | 2.241758 | 0.989829 | 3.231586 | 2.074 | 0.398 |
|  | 102.1099 | 4.858981 | 2.069555 | 0.939965 | 3.00952 | 1.256 | 0.409 |
| Fer | 108.1237 | 5.233557 | 1.943666 | 0.905786 | 2.849452 | 1.194 | 0.32 |
|  | 138.5216 | 5.924379 | 2.149161 | 0.966811 | 3.115972 | 1.433 | 0.366 |
|  | 122.7225 | 5.360647 | 1.800452 | 0.825388 | 2.62584 | 1.566 | 0.393 |
|  | 145.5328 | 5.414926 | 1.968195 | 0.906761 | 2.874956 | 1.702 | 0.4 |
|  | 84.7735 | 4.099856 | 2.011658 | 0.90418 | 2.915838 | 1.764 | 0.402 |
|  | 136.1524 | 5.126881 | 1.936983 | 0.874653 | 2.811636 | 1.789 | 0.428 |
|  | 125.5364 | 4.862259 | 1.779101 | 0.827689 | 2.60679 | 1.873 | 0.439 |
|  | 93.29722 | 4.942662 | 1.996443 | 0.885863 | 2.882306 | 2.017 | 0.451 |
|  | 80.40503 | 3.996327 | 2.083091 | 0.910627 | 2.993718 | 2.054 | 0.549 |
|  | 135.9259 | 4.54616 | 1.840681 | 0.847644 | 2.688324 | 2.095 | 0.578 |
|  | 103.8944 | 4.142035 | 1.976851 | 0.895201 | 2.872053 | 1.397 | 0.435 |
|  | 104.8143 | 4.273937 | 1.240231 | 0.630122 | 1.870353 | 1.494 | 0.33 |
|  | 121.7517 | 4.355118 | 1.899405 | 0.857334 | 2.75674 | 1.948 | 0.533 |
|  | 139.0062 | 4.408985 | 1.913834 | 0.861837 | 2.775671 | 1.896 | 0.446 |
|  | 147.7354 | 5.834107 | 2.240338 | 1.011715 | 3.252053 | 1.591 | 0.446 |
|  | 99.11046 | 5.404292 | 2.574888 | 1.144071 | 3.718959 | 1.372 | 0.576 |
|  | 117.5358 | 5.083669 | 1.97025 | 0.909942 | 2.880191 | 1.678 | 0.432 |
|  | 85.39858 | 4.965531 | 2.102487 | 0.950042 | 3.052529 | 1.763 | 0.54 |
|  | 123.1012 | 4.852946 | 2.653622 | 1.121393 | 3.775015 | 1.647 | 0.501 |
|  | 131.1664 | 4.58611 | 1.924313 | 0.890002 | 2.814315 | 1.23 | 0.415 |
| H2O | 79.99266 | 4.374544 | 2.765762 | 1.181828 | 3.947589 | 1.337 | 0.498 |
|  | 99.97649 | 4.75503 | 2.668912 | 1.180802 | 3.849715 | 1.596 | 0.367 |
|  | 69.54035 | 4.507803 | 3.118377 | 1.295976 | 4.414354 | 1.841 | 0.39 |
|  | 91.15572 | 4.273718 | 3.411764 | 1.469443 | 4.881207 | 1.314 | 0.382 |
|  | 100.2039 | 5.055995 | 2.265757 | 0.980715 | 3.246472 | 1.507 | 0.435 |
|  | 78.48779 | 4.324375 | 1.866296 | 0.84338 | 2.709676 | 1.882 | 0.432 |
|  | 87.57027 | 5.132793 | 2.702993 | 1.129026 | 3.832019 | 1.477 | 0.528 |
|  | 114.3864 | 4.000921 | 2.375296 | 1.070292 | 3.445589 | 2.013 | 0.518 |
|  | 85.88793 | 5.148009 | 2.11648 | 0.925231 | 3.04171 | 1.197 | 0.399 |
|  | 78.14755 | 4.457052 | 1.766135 | 0.797353 | 2.563488 | 1.209 | 0.461 |
|  | 121.3871 | 4.892162 | 2.085072 | 0.940829 | 3.025901 | 2.032 | 0.346 |
|  | 65.85962 | 3.550247 | 2.047801 | 0.905446 | 2.953248 | 1.642 | 0.354 |
|  | 103.7435 | 3.811109 | 2.080061 | 0.94002 | 3.02008 | 1.248 | 0.35 |
|  | 89.69351 | 3.977866 | 1.975362 | 0.890257 | 2.865618 | 1.59 | 0.558 |
|  | 106.2921 | 4.463512 | 2.138796 | 0.933053 | 3.071849 | 1.441 | 0.366 |
|  | 73.58524 | 2.531982 | 1.666788 | 0.789302 | 2.45609 | 2.054 | 0.348 |
|  | 89.99786 | 4.240265 | 1.940253 | 0.864477 | 2.80473 | 1.393 | 0.344 |
|  | 100.5804 | 4.440545 | 2.00622 | 0.902922 | 2.909143 | 1.301 | 0.5 |
|  | 115.0065 | 4.711491 | 2.216525 | 0.943588 | 3.160113 | 1.477 | 0.333 |
|  | 95.18238 | 4.033036 | 1.958179 | 0.867218 | 2.825398 | 1.794 | 0.407 |
