# Supplementary material for: Effects of a compound Trichoderma agent on Coptis chinensis growth, nutrients, enzyme activity, and microbial community of rhizosphere soil
Source: PeerJ. 2023 Jul 12;11:e15652. doi: 10.7717/peerj.15652 (PMC10349559; doi:10.7717/peerj.15652)
Supplement: Supplemental Information 2 — Raw data for Table 2. [file peerj-11-15652-s002.docx]

| NO. | Organic matter  (g/kg) | Hydrolyzable nitrogen  (mg/kg) | Available phosphorus  (mg/kg) | Available potassium  (mg/kg) | pH |
| --- | --- | --- | --- | --- | --- |
| CTA-1 | 22.79 | 148.62 | 49.16 | 251.53 | 5.15 |
| CTA-2 | 22.33 | 144.72 | 42.01 | 255.33 | 5.04 |
| CTA-3 | 21.70 | 158.88 | 42.55 | 258.39 | 5.24 |
| CTA-4 | 19.68 | 155.19 | 45.33 | 265.00 | 5.16 |
| CTA-5 | 20.31 | 155.51 | 47.94 | 254.19 | 5.05 |
| Fer-1 | 21.62 | 285.41 | 42.37 | 336.17 | 4.37 |
| Fer-2 | 21.58 | 234.45 | 49.23 | 306.10 | 4.44 |
| Fer-3 | 21.15 | 263.10 | 48.48 | 309.36 | 4.49 |
| Fer-4 | 21.52 | 263.74 | 47.12 | 313.10 | 4.34 |
| Fer-5 | 21.73 | 260.46 | 64.37 | 311.94 | 4.35 |
| H2O-1 | 21.26 | 158.64 | 37.63 | 242.54 | 5.13 |
| H2O-2 | 24.01 | 151.48 | 56.02 | 258.89 | 5.01 |
| H2O-3 | 21.42 | 155.54 | 43.73 | 257.27 | 5.05 |
| H2O-4 | 20.49 | 146.84 | 45.63 | 251.31 | 5 |
| H2O-5 | 20.80 | 146.54 | 41.70 | 254.27 | 4.99 |
