# Supplementary material for: Effects of a compound Trichoderma agent on Coptis chinensis growth, nutrients, enzyme activity, and microbial community of rhizosphere soil
Source: PeerJ. 2023 Jul 12;11:e15652. doi: 10.7717/peerj.15652 (PMC10349559; doi:10.7717/peerj.15652)
Supplement: Supplemental Information 3 — Raw data for Table 3. [file peerj-11-15652-s003.docx]

| NO. | Urease  （μg/d /g ） | Sucrase  (mg/d/g ) | Catalase（mmol/d/g ） | Alkaline phosphatase  （μmol/d/g） |
| --- | --- | --- | --- | --- |
| CTA-1 | 606.31 | 6.77 | 25.38 | 1.36 |
| CTA-2 | 505.83 | 7.00 | 24.38 | 1.20 |
| CTA-3 | 599.57 | 6.32 | 25.93 | 1.76 |
| CTA-4 | 591.38 | 6.33 | 25.30 | 1.25 |
| CTA-5 | 622.53 | 6.42 | 25.83 | 1.81 |
| Fer-1 | 231.39 | 6.86 | 17.81 | 0.74 |
| Fer-2 | 198.66 | 6.76 | 17.34 | 0.64 |
| Fer-3 | 202.82 | 6.23 | 17.24 | 0.97 |
| Fer-4 | 225.93 | 6.07 | 19.86 | 0.62 |
| Fer-5 | 215.60 | 5.44 | 19.05 | 0.77 |
| H2O-1 | 523.78 | 5.65 | 22.58 | 1.35 |
| H2O-2 | 499.52 | 6.01 | 24.29 | 1.23 |
| H2O-3 | 510.43 | 6.71 | 23.36 | 1.31 |
| H2O-4 | 520.33 | 5.77 | 22.87 | 1.05 |
| H2O-5 | 501.67 | 5.64 | 23.13 | 1.15 |
