# Supplementary material for: Effects of a compound Trichoderma agent on Coptis chinensis growth, nutrients, enzyme activity, and microbial community of rhizosphere soil
Source: PeerJ. 2023 Jul 12;11:e15652. doi: 10.7717/peerj.15652 (PMC10349559; doi:10.7717/peerj.15652)
Supplement: Supplemental Information 4 — Raw data for Table 4. [file peerj-11-15652-s004.docx]

|  | Treatment | Observed  species | Shannon | Simpson | Chao1 | ACE |
| --- | --- | --- | --- | --- | --- | --- |
| Fungus | CTA | 1128 | 5.151 | 0.873 | 1297.235 | 1347.559 |
|  | CTA | 722 | 3.809 | 0.735 | 785.02 | 819.517 |
|  | CTA | 900 | 4.602 | 0.822 | 1052.468 | 1069.476 |
|  | CTA | 1070 | 4.715 | 0.808 | 1221.181 | 1271.252 |
|  | CTA | 1068 | 4.528 | 0.788 | 1228.722 | 1268.073 |
|  | Fer | 1371 | 6.874 | 0.971 | 1514.519 | 1532.651 |
|  | Fer | 1323 | 6.425 | 0.952 | 1488.976 | 1488.687 |
|  | Fer | 926 | 5.77 | 0.952 | 1031.225 | 1051.78 |
|  | Fer | 1383 | 7.236 | 0.981 | 1496.569 | 1516.738 |
|  | Fer | 1241 | 6.398 | 0.956 | 1411.946 | 1448.801 |
|  | H2O | 1325 | 6.523 | 0.96 | 1501.254 | 1526.127 |
|  | H2O | 1399 | 6.658 | 0.968 | 1562.989 | 1619.426 |
|  | H2O | 1266 | 6.476 | 0.967 | 1413.248 | 1463.137 |
|  | H2O | 1129 | 6.456 | 0.971 | 1282.91 | 1309.391 |
|  | H2O | 1176 | 6.724 | 0.97 | 1207.576 | 1249.483 |
| Bacterium | CTA | 2383 | 8.557 | 0.99 | 2590.07 | 2630.528 |
|  | CTA | 2369 | 8.481 | 0.991 | 2619.075 | 2644.618 |
|  | CTA | 2648 | 8.771 | 0.993 | 2919.985 | 2963.946 |
|  | CTA | 2483 | 8.638 | 0.992 | 2734.51 | 2743.89 |
|  | CTA | 2487 | 8.674 | 0.992 | 2676.095 | 2756.262 |
|  | Fer | 2494 | 8.987 | 0.994 | 2722.52 | 2757.047 |
|  | Fer | 2412 | 8.573 | 0.99 | 2699.018 | 2722.465 |
|  | Fer | 2404 | 8.748 | 0.993 | 2633.867 | 2681.387 |
|  | Fer | 2547 | 9.016 | 0.994 | 2771.19 | 2804.264 |
|  | Fer | 2468 | 8.824 | 0.993 | 2707.059 | 2748.118 |
|  | H2O | 2551 | 8.866 | 0.993 | 2799.986 | 2853.329 |
|  | H2O | 2444 | 8.815 | 0.994 | 2658.987 | 2696.964 |
|  | H2O | 2794 | 8.855 | 0.993 | 4966.195 | 3707.384 |
|  | H2O | 2595 | 8.981 | 0.994 | 2801.651 | 2851.37 |
|  | H2O | 2612 | 8.957 | 0.994 | 2877.014 | 2919.964 |
