# Supplementary material for: Effects of a compound Trichoderma agent on Coptis chinensis growth, nutrients, enzyme activity, and microbial community of rhizosphere soil
Source: PeerJ. 2023 Jul 12;11:e15652. doi: 10.7717/peerj.15652 (PMC10349559; doi:10.7717/peerj.15652)
Supplement: Supplemental Information 5 — Raw data for Table 5. [file peerj-11-15652-s005.docx]

|  |  | Treatment | CTA-1 | CTA-2 | CTA-3 | CTA-4 | CTA-5 | Fer-1 | Fer-2 | Fer-3 | Fer-4 | Fer-5 | H2O-1 | H2O-2 | H2O-3 | H2O-4 | H2O-5 |
| --- | --- | --- | --- | --- | --- | --- | --- | --- | --- | --- | --- | --- | --- | --- | --- | --- | --- |
| Bacterium | 1 | delta_proteobacterium_WX81 | 0.00158 | 0.001739 | 0.002254 | 0.001934 | 0.001704 | 0.002875 | 0.003745 | 0.003053 | 0.004437 | 0.002698 | 0.002449 | 0.00181 | 0.002023 | 0.002449 | 0.001881 |
|  | 2 | Ralstonia_pickettii | 0.056526 | 0.040659 | 0.020037 | 0.022504 | 0.038636 | 0.000266 | 0.000248 | 1.77E-05 | 1.77E-05 | 0 | 3.55E-05 | 7.1E-05 | 8.87E-05 | 0 | 1.77E-05 |
|  | 3 | Bradyrhizobium_elkanii | 0.021687 | 0.020232 | 0.020711 | 0.019948 | 0.0167 | 0.022699 | 0.01899 | 0.025024 | 0.021794 | 0.023214 | 0.018457 | 0.020516 | 0.019788 | 0.021261 | 0.02238 |
|  | 4 | Sphingomonas_koreensis | 0.001846 | 0.000958 | 0.000976 | 0.001047 | 0.024616 | 0.001136 | 0.000834 | 0.000923 | 0.001189 | 0.000745 | 0.000905 | 0.001296 | 0.000905 | 0.001012 | 0.001136 |
|  | 5 | Pasteurellaceae_bacterium | 0 | 0.000177 | 0 | 0 | 0 | 1.77E-05 | 0.00039 | 0 | 0 | 8.87E-05 | 0.014021 | 0.0093 | 0.02387 | 0 | 8.87E-05 |
|  | 6 | Staphylococcus_sciuri | 0.000373 | 0.00126 | 0.000976 | 5.32E-05 | 0.000284 | 0.000373 | 0.000515 | 0 | 0.002076 | 0.014198 | 0.00055 | 0.000692 | 0.000319 | 5.32E-05 | 0.000479 |
|  | 7 | Lolium_perenne | 0.000142 | 5.32E-05 | 1.77E-05 | 0.000195 | 0.002414 | 0.00071 | 0.000177 | 0.000106 | 0.000213 | 5.32E-05 | 0 | 0.000124 | 0.000177 | 0.002307 | 0.009974 |
|  | 8 | Caloramator_australicus | 0 | 1.77E-05 | 0 | 3.55E-05 | 0.009193 | 0 | 0 | 0 | 0 | 0 | 0 | 0 | 0 | 0 | 0 |
|  | 9 | bacterium_Ellin6089 | 0.002165 | 0.001686 | 0.002201 | 0.001739 | 0.001402 | 0.002502 | 0.002023 | 0.002502 | 0.002236 | 0.001828 | 0.001757 | 0.002059 | 0.001739 | 0.001863 | 0.001562 |
|  | 10 | Hyphomicrobium_facile | 0.002272 | 0.002325 | 0.001722 | 0.001846 | 0.001722 | 0.001757 | 0.001757 | 0.00268 | 0.001651 | 0.001988 | 0.001775 | 0.002485 | 0.00252 | 0.00158 | 0.002183 |
| Fungus | 1 | Trichoderma_hamatum | 0.324939 | 0.49748 | 0.403506 | 0.424829 | 0.446304 | 0.03555 | 0.017075 | 0.041315 | 0.029145 | 0.063464 | 0.031488 | 0.029886 | 0.02552 | 0.023312 | 0.077708 |
|  | 2 | Fusarium_sp | 0.047231 | 0.051496 | 0.043068 | 0.022166 | 0.022301 | 0.029381 | 0.026582 | 0.033881 | 0.087349 | 0.038938 | 0.037269 | 0.047922 | 0.056317 | 0.015592 | 0.021525 |
|  | 3 | Sporothrix_nigrograna | 0.000185 | 0.000101 | 8.43E-05 | 3.37E-05 | 0.003961 | 0.000944 | 0.181424 | 0.000624 | 0.025841 | 0.004871 | 0.000438 | 0.000523 | 0.000624 | 0.000421 | 0.000152 |
|  | 4 | Trichoderma_sp | 0.071993 | 0.135474 | 0.095558 | 0.097969 | 0.091041 | 0.013603 | 0.006726 | 0.010265 | 0.006507 | 0.01067 | 0.00745 | 0.006793 | 0.003574 | 0.004804 | 0.009642 |
|  | 5 | Fusicolla_merismoides | 0.002242 | 0.001652 | 0.005242 | 0.002276 | 0.003624 | 0.006776 | 0.008428 | 0.123557 | 0.008732 | 0.021323 | 0.006254 | 0.007012 | 0.005917 | 0.003456 | 0.004383 |
|  | 6 | Plectosphaerella_sp | 0.000809 | 0.000219 | 0.000337 | 0.011833 | 0.000775 | 0.112971 | 0.001922 | 0 | 0.001736 | 0.002006 | 0.004265 | 0.003675 | 0.002427 | 0.001197 | 0.000877 |
|  | 7 | Leotia_sp | 0.003759 | 0.004366 | 0.000961 | 0.008647 | 0.005107 | 0.021323 | 0.016216 | 0.013047 | 0.029279 | 0.015542 | 0.00359 | 0.002528 | 0.00381 | 0.000877 | 0.111471 |
|  | 8 | Mortierella_sp | 0.040556 | 0.017783 | 0.029819 | 0.022368 | 0.040556 | 0.030965 | 0.058778 | 0.052811 | 0.037522 | 0.06965 | 0.097649 | 0.076123 | 0.059284 | 0.036865 | 0.018592 |
|  | 9 | Thelephoraceae_sp | 0.034437 | 0.025268 | 0.038972 | 0.015997 | 0.016502 | 0.015306 | 0.014665 | 0.018879 | 0.041197 | 0.045445 | 0.080421 | 0.042798 | 0.045647 | 0.031757 | 0.039393 |
|  | 10 | Trichocladium_griseum | 0.008748 | 0.005596 | 0.006119 | 0.014395 | 0.008664 | 0.011024 | 0.010923 | 0.069987 | 0.009355 | 0.01271 | 0.014968 | 0.051445 | 0.009692 | 0.036629 | 0.04713 |
