# Supplementary material for: Effects of a compound Trichoderma agent on Coptis chinensis growth, nutrients, enzyme activity, and microbial community of rhizosphere soil
Source: PeerJ. 2023 Jul 12;11:e15652. doi: 10.7717/peerj.15652 (PMC10349559; doi:10.7717/peerj.15652)
Supplement: Supplemental Information 8 — Raw data of fungal function prediction according to Mode. [file peerj-11-15652-s008.docx]

| **Trophic_Mode** | **CTA1** | **CTA2** | **CTA3** | **CTA4** | **CTA5** |
| --- | --- | --- | --- | --- | --- |
| **Pathogen-Saprotroph-Symbiotroph** | 0.000321 | 0.000051 | 0.000017 | 0.000237 | 0.000254 |
| **Pathotroph** | 0.021631 | 0.011498 | 0.036245 | 0.046144 | 0.025558 |
| **Pathotroph-Saprotroph** | 0.052410 | 0.052780 | 0.044824 | 0.028305 | 0.028406 |
| **Pathotroph-Saprotroph-Symbiotroph** | 0.022892 | 0.014210 | 0.016488 | 0.020213 | 0.021947 |
| **Pathotroph-Symbiotroph** | 0.000794 | 0.000894 | 0.000744 | 0.001670 | 0.001214 |
| **Saprotroph** | 0.535772 | 0.714339 | 0.618950 | 0.681034 | 0.672304 |
| **Saprotroph-Symbiotroph** | 0.001332 | 0.000557 | 0.000810 | 0.001485 | 0.001315 |
| **Symbiotroph** | 0.045944 | 0.026539 | 0.034448 | 0.032561 | 0.028499 |
| **Unassigned** | 0.319012 | 0.179202 | 0.247551 | 0.188478 | 0.220602 |
| **Trophic_Mode** | **Fer1** | **Fer2** | **Fer3** | **Fer4** | **Fer5** |
| **Pathogen-Saprotroph-Symbiotroph** | 0.000826 | 0.001350 | 0.000101 | 0.002174 | 0.000034 |
| **Pathotroph** | 0.187227 | 0.063505 | 0.017924 | 0.046602 | 0.031193 |
| **Pathotroph-Saprotroph** | 0.060836 | 0.067360 | 0.037306 | 0.121283 | 0.059488 |
| **Pathotroph-Saprotroph-Symbiotroph** | 0.039916 | 0.208445 | 0.011566 | 0.059573 | 0.052287 |
| **Pathotroph-Symbiotroph** | 0.010098 | 0.005733 | 0.002748 | 0.005697 | 0.001502 |
| **Saprotroph** | 0.336078 | 0.303621 | 0.403887 | 0.318378 | 0.367262 |
| **Saprotroph-Symbiotroph** | 0.001688 | 0.001215 | 0.002008 | 0.001433 | 0.001029 |
| **Symbiotroph** | 0.077062 | 0.049533 | 0.105864 | 0.076336 | 0.057862 |
| **Unassigned** | 0.286356 | 0.299339 | 0.418696 | 0.368627 | 0.429469 |
| **Trophic_Mode** | **H2O1** | **H2O2** | **H2O3** | **H2O4** | **H2O5** |
| **Pathogen-Saprotroph-Symbiotroph** | 0.000286 | 0.000320 | 0.000118 | 0.000118 | 0.000051 |
| **Pathotroph** | 0.038998 | 0.037025 | 0.036214 | 0.043782 | 0.037749 |
| **Pathotroph-Saprotroph** | 0.043359 | 0.068694 | 0.062826 | 0.020450 | 0.030264 |
| **Pathotroph-Saprotroph-Symbiotroph** | 0.009003 | 0.039732 | 0.030883 | 0.031000 | 0.022302 |
| **Pathotroph-Symbiotroph** | 0.002683 | 0.003761 | 0.003760 | 0.001433 | 0.003693 |
| **Saprotroph** | 0.301337 | 0.338883 | 0.249106 | 0.272978 | 0.413270 |
| **Saprotroph-Symbiotroph** | 0.001333 | 0.001216 | 0.001403 | 0.001215 | 0.002666 |
| **Symbiotroph** | 0.090578 | 0.075226 | 0.131544 | 0.100490 | 0.074231 |
| **Unassigned** | 0.512545 | 0.435287 | 0.484277 | 0.528656 | 0.415911 |
